# The Fun30 Chromatin Remodeler Fft3 Controls Nuclear Organization and Chromatin Structure of Insulators and Subtelomeres in Fission Yeast

**DOI:** 10.1371/journal.pgen.1005101

**Published:** 2015-03-23

**Authors:** Babett Steglich, Annelie Strålfors, Olga Khorosjutina, Jenna Persson, Agata Smialowska, Jean-Paul Javerzat, Karl Ekwall

**Affiliations:** 1 Department of Biosciences and Nutrition; Center for Innovative Medicine, Karolinska Institutet, Novum Building, Huddinge, Sweden; 2 Univ. Bordeaux, Institut de Biochimie et Génétique Cellulaires, UMR 5095, Bordeaux, France; 3 CNRS, Institut de Biochimie et Génétique Cellulaires, UMR 5095, Bordeaux, France; University of California San Francisco, UNITED STATES

## Abstract

In eukaryotic cells, local chromatin structure and chromatin organization in the nucleus both influence transcriptional regulation. At the local level, the Fun30 chromatin remodeler Fft3 is essential for maintaining proper chromatin structure at centromeres and subtelomeres in fission yeast. Using genome-wide mapping and live cell imaging, we show that this role is linked to controlling nuclear organization of its targets. In *fft3∆* cells, subtelomeres lose their association with the LEM domain protein Man1 at the nuclear periphery and move to the interior of the nucleus. Furthermore, genes in these domains are upregulated and active chromatin marks increase. Fft3 is also enriched at retrotransposon-derived long terminal repeat (LTR) elements and at tRNA genes. In cells lacking Fft3, these sites lose their peripheral positioning and show reduced nucleosome occupancy. We propose that Fft3 has a global role in mediating association between specific chromatin domains and the nuclear envelope.

## Author Summary

In the genome of eukaryotic cells, domains of active and repressive chromatin alternate along the chromosome arms. Insulator elements are necessary to shield these different environments from each other. In the fission yeast *Schizosaccharomyces pombe*, the chromatin remodeler Fft3 is required to maintain the repressed subtelomeric chromatin. Here we show that Fft3 maintains nucleosome structure of insulator elements at the subtelomeric borders. We also observe that subtelomeres and insulator elements move away from the nuclear envelope in cells lacking Fft3. The nuclear periphery is known to harbor repressive chromatin in many eukaryotes and has been implied in insulator function. Our results suggest that chromatin remodeling through Fft3 is required to maintain proper chromatin structure and nuclear organization of insulator elements.

## Introduction

Nuclear architecture, i.e. non-random positioning of chromosomal loci and nuclear components in three dimensions, is important in organizing genome processes such as transcription, DNA replication and DNA repair [1–3]. One crucial aspect of this organization is the interaction between chromatin and the nuclear periphery. In many eukaryotic species, chromosomal domains near the nuclear envelope show low expression levels, repressive chromatin marks and low gene density [4–7]. These domains interact with the nuclear lamina [4,5] or inner nuclear membrane (INM) proteins, such as the LEM-domain proteins [6,7]. How chromosomal loci are targeted to the nuclear envelope is still poorly understood, although step-wise methylation of lysine 9 on histone H3 (H3K9me) [8,9] and interaction with different nuclear membrane proteins [10] are known to contribute.

In the fission yeast *Schizosaccharomyces pombe*, the INM protein Man1 interacts with about a third of the genome, mainly at lowly transcribed genes [7]. Especially striking, we observed Man1 binding to large domains adjacent to the telomeres of chromosomes I and II, which are characterized by a unique type of chromatin [11]. These subtelomeric domains show low levels of histone methylation, in both the repressive mark H3K9me2 and the active mark H3K4me2, as well as lower histone acetylation and H2A.Z levels compared to euchromatin [11,12]. Many genes in these regions are lowly expressed in rapidly growing cells, but are induced during nutritional stress or meiosis [13]. The borders of the subtelomeric domains are bound by the chromatin remodeling factor Fft3 [14]. Maintenance of the special chromatin state inside these domains in rapidly growing cells depends on Fft3, since a deletion of the remodeler results in a strong upregulation of subtelomeric genes and spreading of euchromatin marks into the domains.

Fft3 belongs to a highly conserved subfamily of SNF2 remodeling enzymes. Fft3 homologs are present in all eukaryotes examined, including Fun30 in *S*. *cerevisiae*, ETL1 in mouse and SMARCAD1 in humans [15,16]. Fun30 subfamily enzymes act in regulating chromatin, maintaining silent chromatin domains and preserving genome stability [14,16–22].

Here, we explore the interplay between genome organization and transcriptional regulation. We show that Fft3 maintains chromatin structure and peripheral positioning of subtelomeres by binding to and remodeling nucleosomes at their borders. In addition to the subtelomeres, Fft3 interacts with TFIIIC and Pol III at tRNA genes and affects nucleosome positioning and interaction with the nuclear periphery. We propose that Fft3 maintains nucleosome stability and peripheral positioning of these elements and thereby preserves proper genome organization.

## Results

### Deletion of *fft3* results in altered chromatin properties and decreased nuclear envelope interactions at subtelomeres

**Fig 1:**
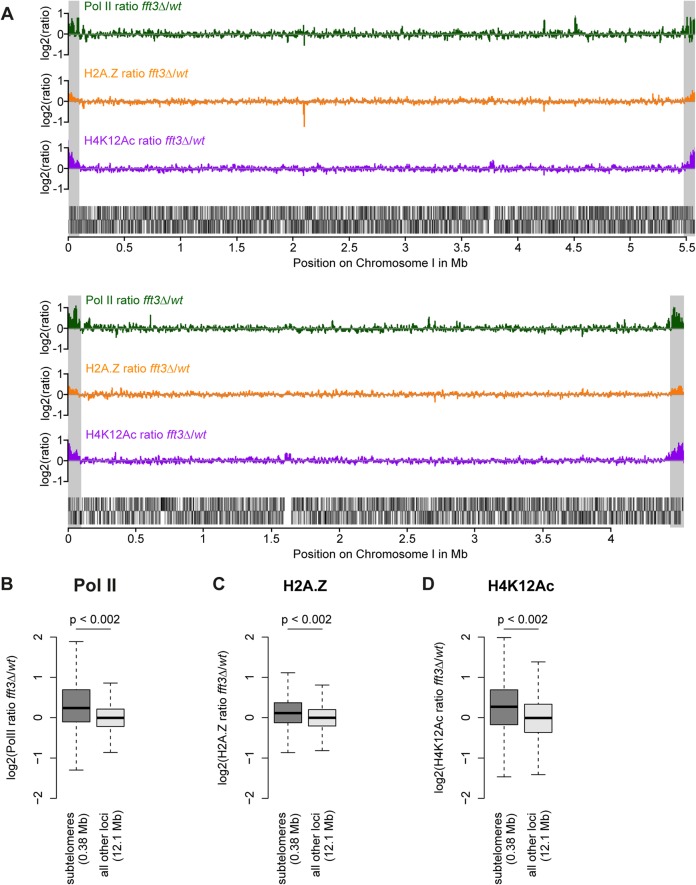
Chromatin changes in *fft3*Δ cells are pronounced at the subtelomeres of chromosomes I and II. **(A)** Subtelomeric regions show strong changes in Pol II and H2A.Z occupancy, as well as H4K12Ac levels. H2A.Z and H4K12Ac ChIP-chip data from [14]. Subtelomeric regions are marked in gray. **(B-D)** Pol II, H2A.Z and H4K12Ac are significantly enriched over subtelomeric regions of chromosomes I and II in *fft3Δ* cells compared to wild type cells.

We previously showed that subtelomeric genes are upregulated in cells lacking the chromatin remodeler Fft3 [14]. To see whether these expression changes coincide with an altered chromatin structure, we performed genome-wide ChIP-chip for three hallmarks of active chromatin: RNA polymerase II (Pol II), the histone variant H2A.Z and the histone modification H4K12Ac. All three marks show a striking increase over the subtelomeric chromatin domains on chromosomes I and II (Fig. 1A) in *fft3Δ* cells compared to wild type, allowing for a chromatin structure more permissive to transcription. These increases are significant when compared with the rest of the genome (p<0.002, Fig. 1 B-D) and could be verified by ChIP qPCR (S1 Fig). Based on these observations, we conclude that Fft3 affects both expression levels and chromatin properties at the subtelomeres.

All data is shown as boxplot, with p-values calculated by circular permutation test (see Materials and Methods).

**Fig 2:**
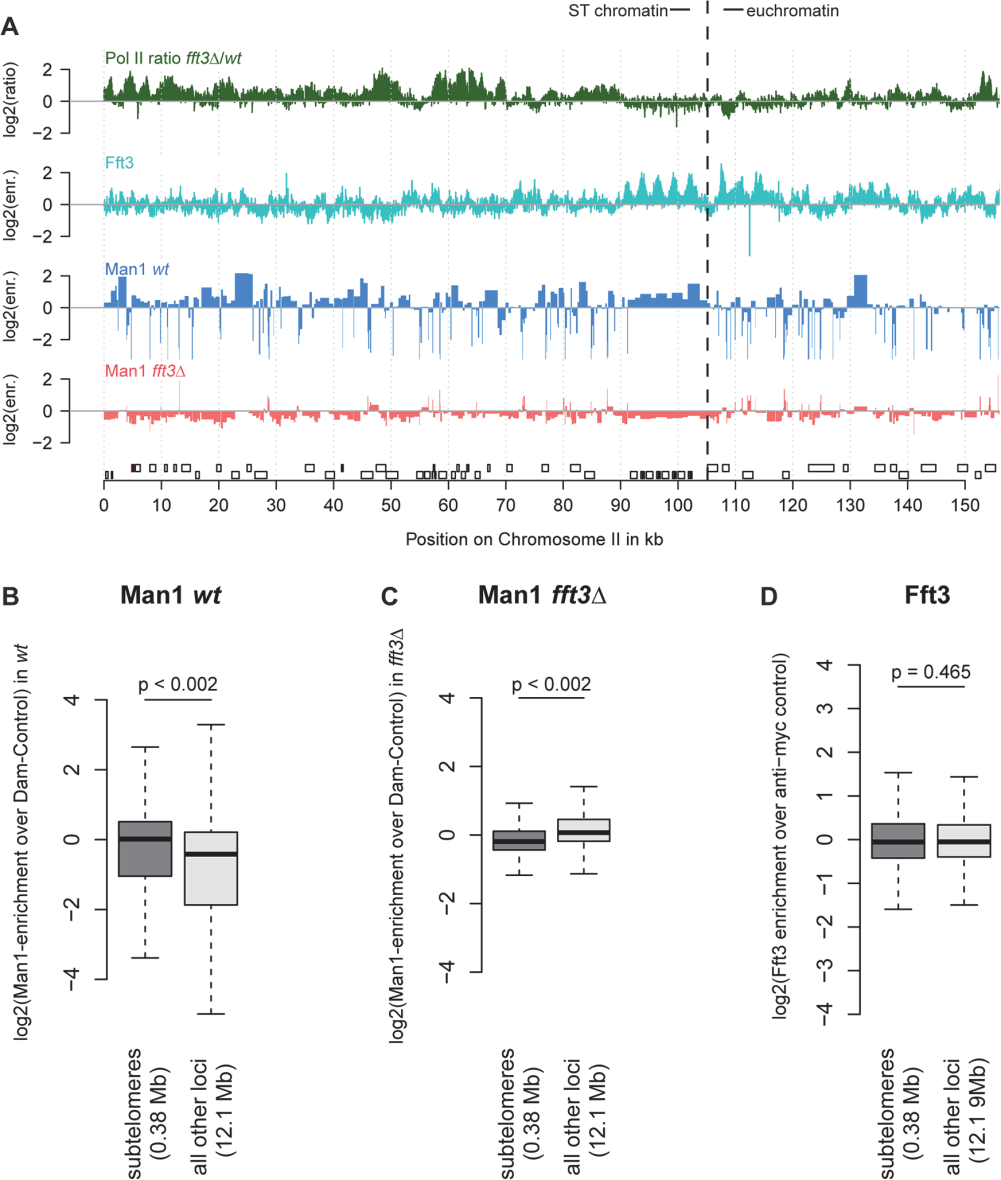
Chromatin changes at subtelomeres in *fft3Δ* cells coincide with changes in nuclear organization. **(A)** Association with the nuclear envelope in wild type is lost in *fft3Δ* cells. Shown over the left subtelomere of chromosome II are log2-ratios of Pol II enrichment in *fft3Δ vs*. wt, log2-enrichment of Fft3-myc ChIP over anti-myc control ChIP, log2-enrichment Man1-Dam over the Dam only control in wild type, log2-enrichment Man1-Dam over Dam only control in *fft3Δ* cells. **(B)** Subtelomeric chromatin is associated with the nuclear periphery in wild type cells. Man1-Dam enrichment over Dam-only control is shown as boxplot. **(C)** Association of the subtelomeres with the nuclear periphery is lost in *fft3Δ* cells. Man1-Dam enrichment over Dam-only control is shown as boxplot. **(D)** Fft3 is not enriched at subtelomeres. Fft3-enrichment over anti-myc control is shown as boxplot.

When we mapped interactions between the INM protein Man1 and chromatin, we observed that subtelomeres are strongly enriched for Man1 [7]. We hypothesized that the expression and chromatin changes in *fft3Δ* cells might be accompanied by an altered nuclear organization. Indeed, DamID mapping revealed that Man1 interactions with the subtelomeres are strongly reduced in *fft3* ***Δ*** cells (Fig. 2A-C, S2 Fig). Interestingly, Fft3 itself does not bind the subtelomeric domains, only their borders (Fig. 2A,D).

Taken together, these results suggest that deleting *fft3* leads to drastic changes in chromatin composition and positioning of these large domains while interacting only with the domain borders.

### Fft3 and Bqt4 cooperate in anchoring subtelomeres to the nuclear envelope

**Fig 3:**
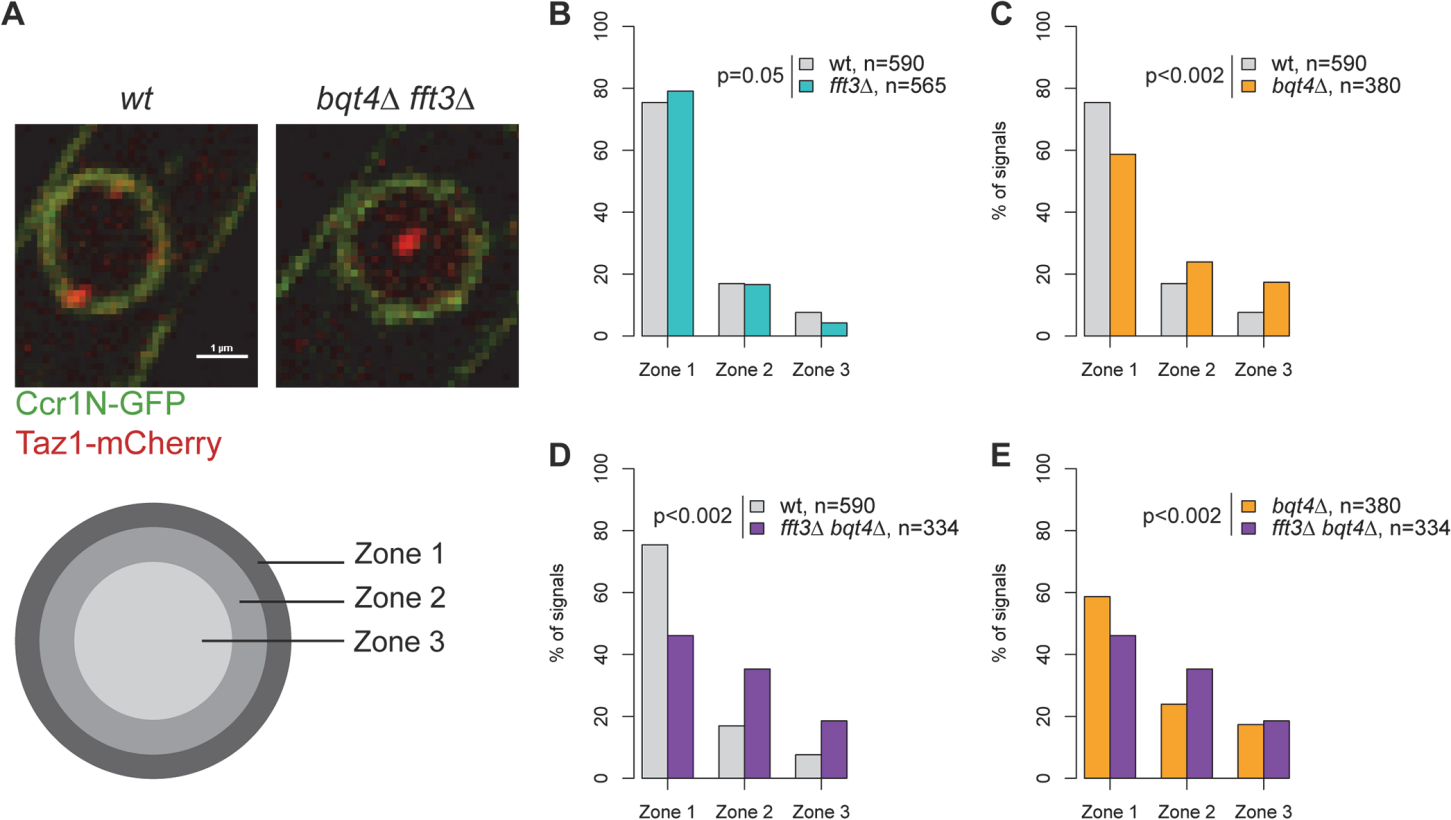
Fft3 and Bqt4 anchor subtelomeres at the nuclear envelope. **(A)** Live cell microscopy examples for wild type and *bqt4Δ fft3Δ* cells. The nuclear envelope is stained by Ccr1N-GFP (green) and the telomeres by Taz1-mCherry (red). Cells were scored as belonging to either of three equal volume zones depending on the distance between the nuclear envelope and the telomere signal. **(B-E)** Telomeres are released from the nuclear envelope in *bqt4Δ* cells, but move even further into the nuclear interior in *bqt4Δ fft3Δ* cells, where subtelomeres lose association with the nuclear envelope. Percentages of cells in each of the zones in different strains are shown, with number of cells measured in the legend. Significance testing was done using a two-sided Chi-square test.

Our data suggest that Man1 and Fft3 have essential roles in tethering subtelomeres to the NE. To explore this further, we monitored the intranuclear position of the telomeres in live cells using the telomere associated protein Taz1. Based on their relative distance to the nuclear envelope, the Taz1 signals in each cell were assigned to one of three zones of equal volume (Fig. 3A). As expected, about 75% of telomere signals are localized in the outermost zone, close to the nuclear envelope, in wild type cells (Fig. 3B). We observed no change in telomere localization in *fft3Δ* cells (Fig. 3B), suggesting that the telomeres themselves are unaffected by the changes in the subtelomeres. The anchoring of telomeres to NE has been shown to depend on the Bqt4 protein [23]. Indeed, we observed that deleting *bqt4* reduced the number of telomeres in zone 1 to below 60%, with an increased number of cells showing telomere signals in the inner zones (Fig. 3C). The loss of peripheral localization is exacerbated in cells lacking both Bqt4 and Fft3 (Fig. 3D-E), with more than 50% of the cells now showing telomere signals in the two innermost zones. We obtained similar results using fluorescence in situ hybridization (FISH, S3 Fig). Signals from a subtelomeric FISH probe showed a modest shift towards the interior in *fft3Δ* cells compared to wildtype. In *fft3Δ bqt4Δ* cells, the majority of FISH signals were found in the two innermost zones, while deletion of *bqt4* by itself had no effect on localization. Taken together, these results demonstrate that Bqt4 and Fft3 work together in anchoring the subtelomeres to the nuclear envelope, with Bqt4 attaching the telomere end and Fft3 preserving the interaction with Man1 over the subtelomeres.

### Fft3 interacts with solo LTRs at subtelomeric borders and genome-wide

**Fig 4:**
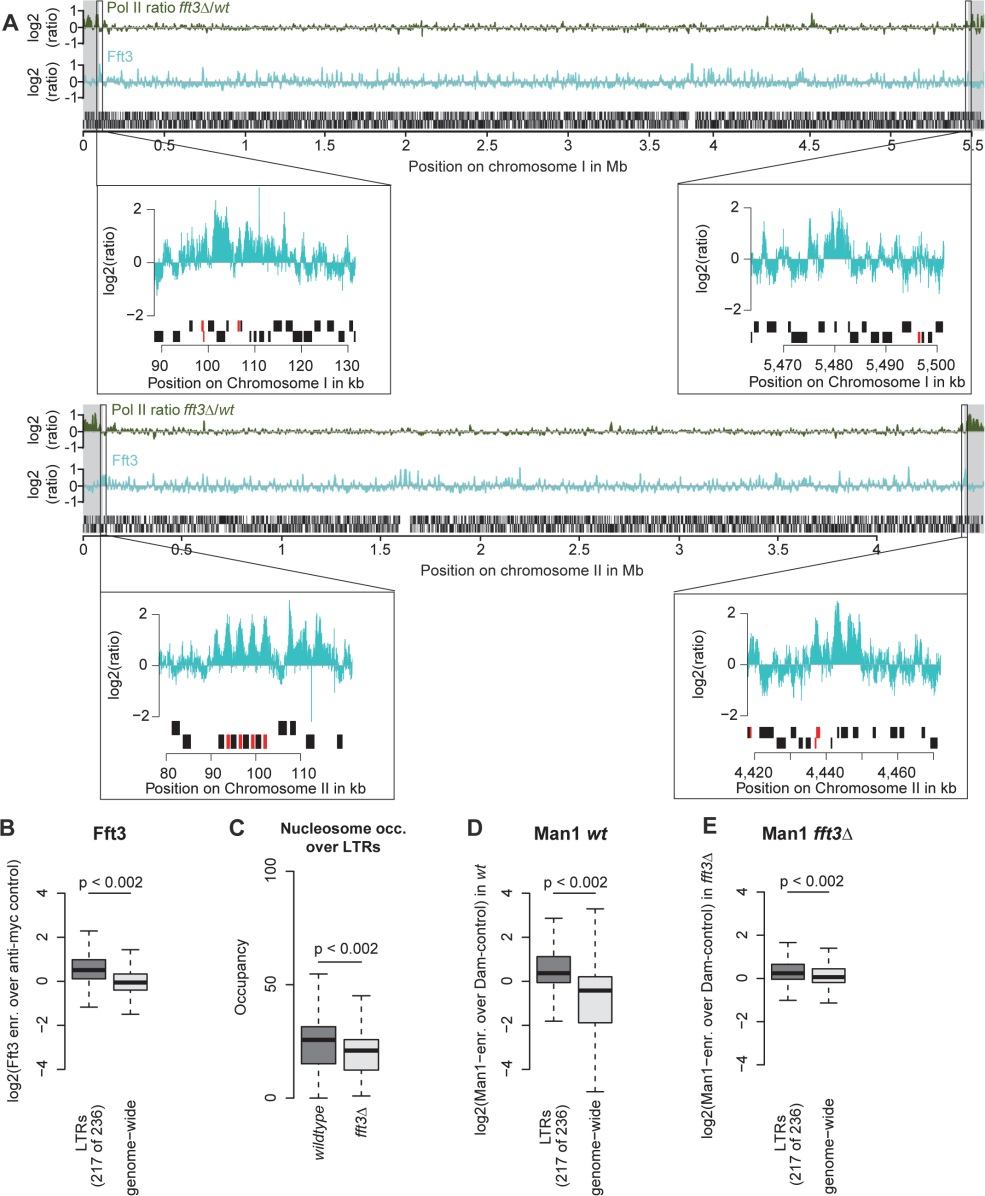
Fft3 binds LTR elements at subtelomeric borders and genome-wide, affecting nucleosome occupancy and peripheral positioning. **(A)** Fft3 binding at subtelomeric borders occurs over or in close proximity to LTR elements. Graphs show Pol II enrichment in *fft3Δ* compared to wild type (green) and Fft3 enrichment compared to anti-myc control (blue) over chromosomes I and II. Subtelomeric regions are marked in gray and small panels show Fft3 binding at subtelomeric borders, with LTR elements marked in red. **(B)** Fft3 interacts with LTRs genome-wide. Fft3-enrichment over anti-myc control is shown as boxplot. **(C)** Nucleosome occupancy at LTRs is reduced in *fft3Δ* cells. The average number of reads mapping to each LTR is shown for wild type and *fft3Δ*. P-value was obtained using paired, two-sided Mann-Whitney U test. **(D)** LTRs interact with Man1 at the nuclear periphery. Man1-Dam enrichment over Dam-only control is shown as boxplot. **(E)** Interaction with Man1 is reduced in *fft3Δ* cells. Man1-Dam enrichment over Dam-only control is shown as boxplot.

We next asked what marks the subtelomeric borders aside from Fft3 binding. Interestingly, all four borders feature long terminal repeat elements (LTRs) either at the Fft3 binding sites or in close proximity (Fig. 4A). Assuming that this is a sequence feature that Fft3 recognizes, we looked at all LTRs in the *S*. *pombe* genome and found that Fft3 is strongly enriched over these elements genome-wide (Fig. 4B). To study how Fft3 affects nucleosome positioning and occupancy, we performed micrococcal nuclease digestion followed by sequencing (MNase-seq). Interestingly, we observed a significant decrease in nucleosome occupancy over LTR elements in *fft3Δ* cells compared to wild type (Fig. 4C). This suggests that Fft3 is required for either positioning or maintaining a nucleosome over LTRs.

Since Fft3 alters interactions with the nuclear envelope at subtelomeres, we wondered whether LTRs are affected similarly. In wild type cells, LTR elements are enriched for binding of both Man1 and the nucleoporin Nup85 [24] (Fig. 4D, S4A Fig). Binding for both is slightly reduced when Fft3 is deleted (Fig. 4E, S4B Fig), but still higher than at other loci in the genome in the case of Man1. When we looked at the LTRs in the border of subtelomere IIL specifically, we observed a marked decrease in Man1 association (S4C Fig), suggesting that LTRs in the subtelomeric borders lose their interaction with the nuclear envelope. We wondered whether inserting an LTR into a locus is sufficient to recruit Fft3, but found no increase in Fft3-myc occupancy by ChIP adjacent to the inserted LTR (S4D Fig). This argues against a sequence-specific recognition of LTRs by Fft3 and rather suggests that other factors such as local chromatin structure are necessary for recruitment. In summary, we conclude that Fft3 binds to LTR elements, affecting their nucleosome occupancy and—in part—their peripheral positioning.

### Catalytic activity of Fft3 is required for subtelomeric silencing

**Fig 5:**
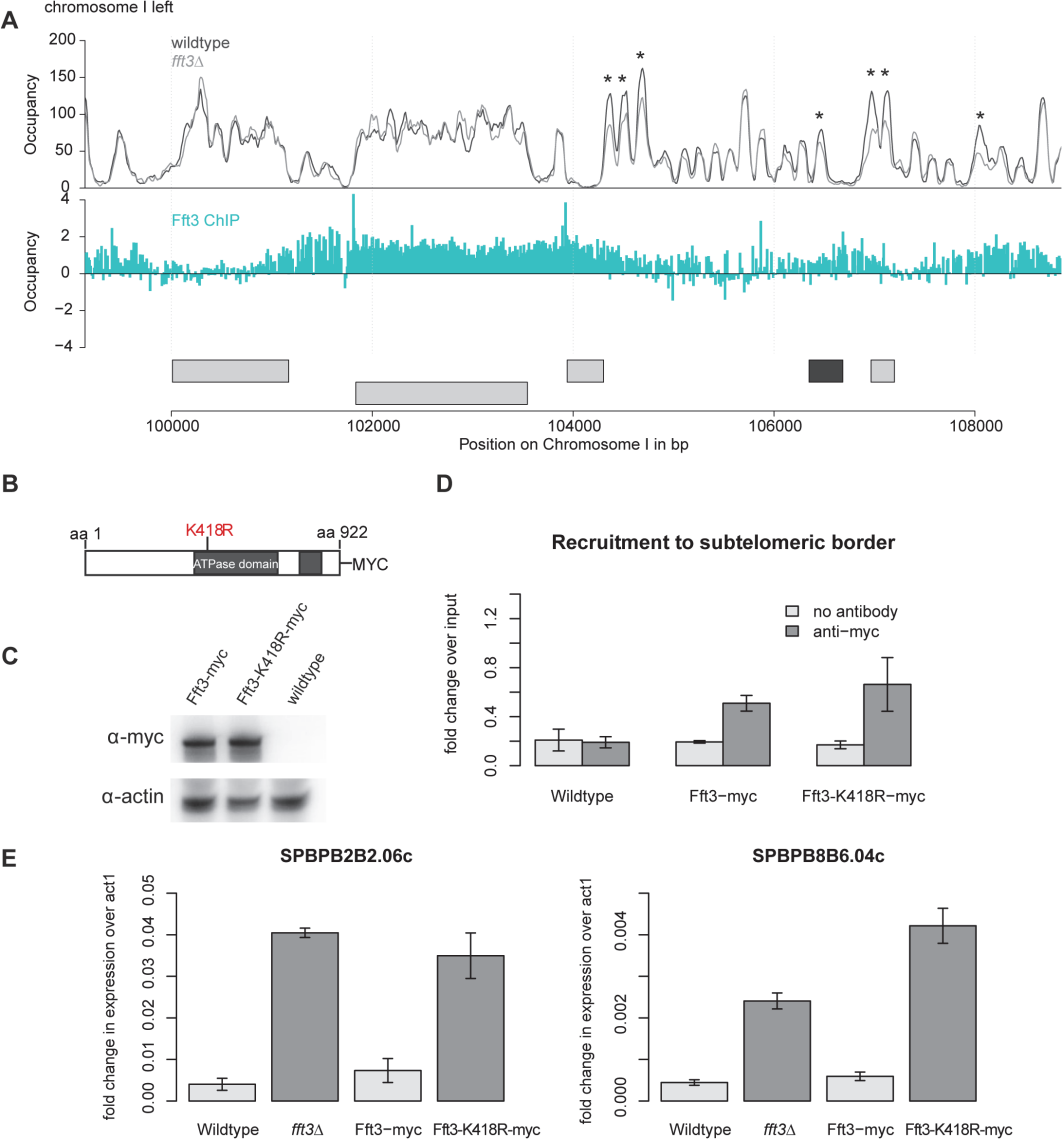
Subtelomeric silencing requires remodeling activity of Fft3. **(A)** Nucleosome occupancy at subtelomeric borders is affected by Fft3. Map shows nucleosome occupancy profiles over the border of the left subtelomere on chromosome I (top panel) and Fft3 enrichment (blue). Genes are shown in light grey, LTRs in dark grey. Asterisks mark positions were nucleosome occupancy is reduced. **(B)** Schematic representation of the Fft3 ATPase mutant containing a point mutation in the ATPase domain that replaces lysine at position 418 (AAA) by arginine (AGA) and a C-terminal Myc-tag. The split ATPase domain is highlighted in grey [16]. **(C)** The Fft3 ATPase mutant (*fft3-K418R*) is expressed at similar levels as the wild type protein (Fft3-myc). Whole-cell lysate was immunoblotted with anti-myc antibodies. A non-tagged wild type strain was included as a control for the specificity of the antibody and actin blot confirms equal loading. **(D)** The Fft3 ATPase mutant is recruited to the LTR elements of the subtelomeric border. Data from duplicate ChIP-qPCR of non-tagged wild type, Fft3-myc and Fft3-K418R-myc is shown as fold difference in enrichment at LTR over *dg*, normalized to input. Error bars represent the standard deviation. **(E)** The Fft3 ATPase mutant shows an upregulation of subtelomeric genes. Total RNA was extracted in duplicate and reversely transcribed into cDNA. The expression levels of two subtelomeric genes were measured with qPCR. Error bars represent the standard deviation.

This raises the question how Fft3 affects chromatin structure at subtelomeric borders. MNase-seq data shows several changes in nucleosome occupancy close to the Fft3 binding sites at the borders (Fig. 5A, S5 Fig). In most cases, nucleosome occupancy was reduced in *fft3Δ* cells compared to wild type, suggesting that loss of Fft3 leads to reduced nucleosome stability in these regions. Based on these observations, we hypothesized that the catalytic activity of Fft3 is essential for its functions in subtelomeric chromatin regulation. We therefore constructed a variant of Fft3 with a point mutation in the ATPase domain (*fft3-K418R*, Fig. 5B), which is known to lead to loss of enzymatic activity [25,26].

The resulting catalytically inactive variant of Fft3 is expressed at similar levels (Fig. 5C) and recruited to the same targets as unmodified Fft3 (Fig. 5D, S6A–C Fig). However, *fft3-K418R* cells mimic the phenotype observed in *fft3Δ*cells: they grow slightly slower than wild type cells at 30°C and show severe growth defects at 25°C and 37°C (S6D Fig). Importantly, subtelomeric genes are upregulated in cells carrying the point mutation in Fft3 (Fig. 5E), as observed in cells lacking Fft3. Furthermore, we observed similar increases in PolII, H3K9Ac and H2A.Z occupancy in *fft3-K418R* and *fft3Δ*cells (S7A–C Fig). As in *fft3Δ* cells, Man1-interaction is reduced in *fft3-K418R* cells compared to wild-type (S7D Fig). Together, these results strongly suggest that ATP-dependent remodeling by Fft3 is directly required to maintain silencing of subtelomeric genes and chromatin structure at the boundaries to the subtelomeres.

### Fft3 colocalizes with the Pol III machinery and TFIIIC

**Fig 6:**
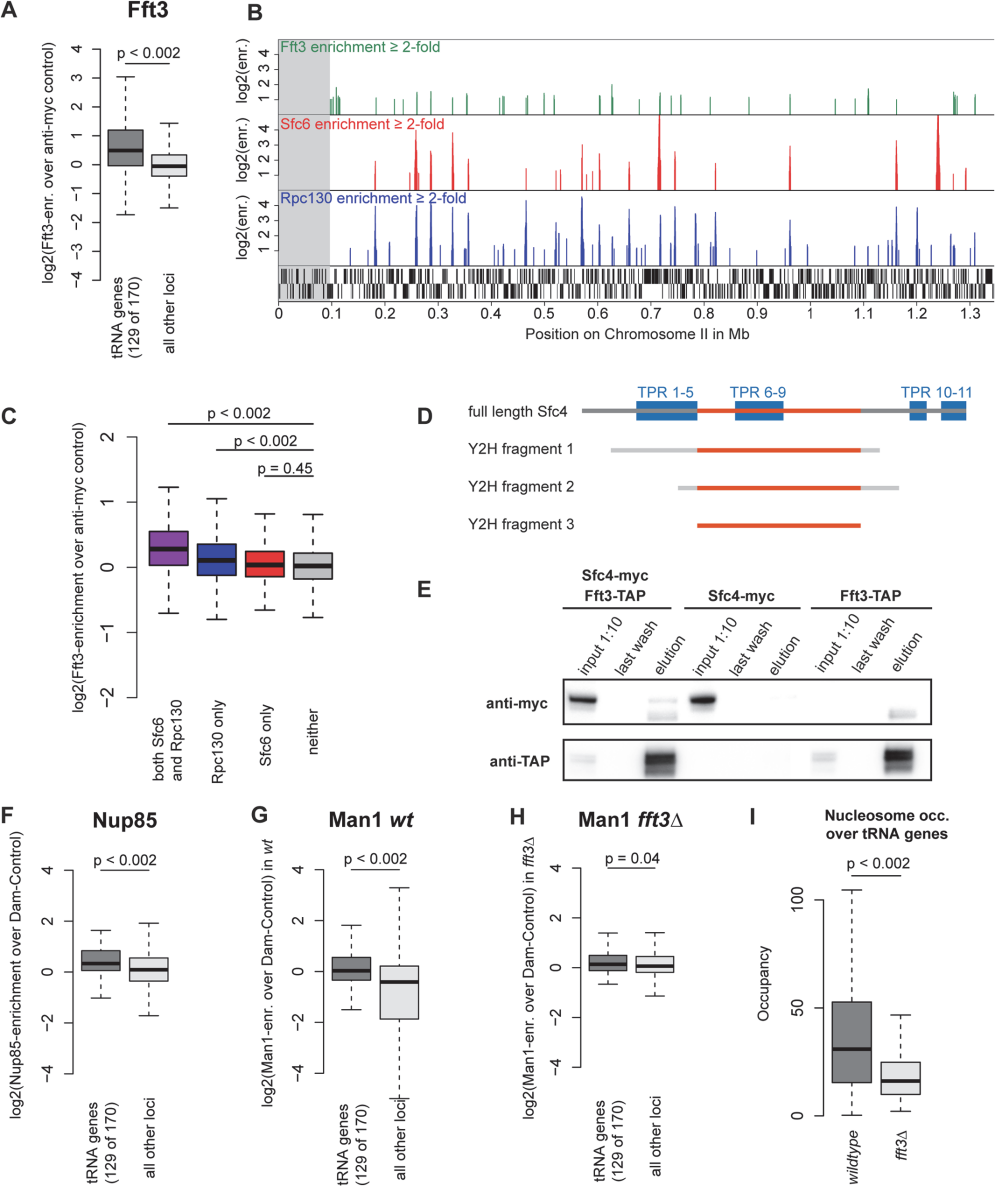
Fft3 interacts with TFIIIC at Pol III transcribed loci and affects peripheral localization of tRNA genes. **(A)** Fft3 is significantly enriched at tRNA genes. Fft3-enrichment over anti-myc control is shown as boxplot. Fft3-myc ChIP-chip data from [14]. **(B)** Fft3-myc binds to loci also bound by the TFIIIC-subunit Sfc6 and the Pol III-component Rpc130. Shown are ChIP-enrichments over 2-fold over input on 1.5 Mb on the left arm of chromosome 2. The subtelomeric chromatin is marked in gray. Fft3-myc ChIP-chip data from [14], Sfc6 and Rpc130 ChIP-chip data from [28]. **(C)** Fft3 is significantly enriched at loci bound by TFIIIC and Pol III together and Pol III alone, but not at loci bound by TFIIIC alone. Fft3-enrichment over input is shown as boxplot. Targets were defined as probes with more than two-fold enrichment (both Sfc6 and Rpc130: 896 probes (53.8 kb), Rpc130 only: 1656 probes (99.3 kb), Sfc6 only: 750 probes (45 kb), neither: 37917 (2,275 kb)). **(D)** Fft3 and Sfc4 interact in a Yeast-2-hybrid screen. Bait fragments of Sfc4 are shown aligned to full-length Sfc4, with overlap between them marked in red. **(E)** Sfc4 is co-purified in a Fft3-TAP purification. Shown are input (diluted to 10%), final wash and eluate for the double-tagged strain (Sfc4-Myc Fft3-TAP) and the two single tagged strains (Sfc4-Myc and Fft3-TAP). **(F)** Nup85 is significantly enriched at tRNA genes. Nup85-Dam enrichment over Dam-only control is shown as boxplot. Nup85-Dam data from [24]. **(G)** The INM protein Man1 is enriched at tRNA genes in wild type *S*. *pombe*. Man1-Dam enrichment over Dam-only control is shown as boxplot. **(H)** Man1 is no longer enriched at tRNA genes in *fft3Δ* cells. Man1-Dam enrichment over Dam-only control in *fft3Δ* cells is shown as boxplot. **(I)** Nucleosome occupancy at tRNA genes is reduced in *fft3Δ* cells. The average number of reads mapping to each tRNA is shown for wild type and *fft3Δ*. P-value was obtained using paired, two-sided Mann-Whitney U test.

After observing the function of Fft3 at subtelomeres and LTR, we set out to explore whether Fft3 also plays a role in regulating chromatin structure elsewhere in the genome. Among other features, we found a preference of Fft3 for snRNA genes, snoRNA genes and replication origins (S8A Fig). Most prominently, we observed a significant enrichment of Fft3 at tRNA genes (Fig. 6A) compared to the rest of the genome. tRNA genes are transcribed by the RNA polymerase III (Pol III) machinery and require the transcription factor TFIIIC for recruitment of Pol III (reviewed in [27]). We also observed Fft3 enrichment at 5S rRNA genes, which are transcribed by Pol III (S8B Fig). When we compared the Fft3 binding profile to Pol III and TFIIIC maps [28], we found a strong overlap in binding sites (Fig. 6B). Several reports have identified loci called *ETC* (Extra-TFIIIC) or *COC* (chromosome-organizing clamps) sites across the genome that are occupied by TFIIIC but not by any other components of the Pol III machinery [28–30]. While Fft3 binding coincides with Pol III binding, we did not observe enrichment of Fft3 at *ETC*/*COC* sites (Fig. 6C). This suggests that Fft3 binds to actively transcribed Pol III sites.

To search for interaction partners of Fft3, we carried out a yeast two-hybrid screen using the full-length Fft3 protein as the bait against an *S*. *pombe* cDNA library. This screen identified several cDNAs encoding Sfc4 (Fig. 6D), a subunit of TFIIIC and homologous to *S*. *cerevisiae* Tfc4p and human TFIIIC102 [31]. The clones cover a set of tetratricopeptide repeats (TPR) which function as sites for protein-protein interactions [32], suggesting that Fft3 could interact with Sfc4 through this domain. To examine whether Fft3 and Sfc4 also interact *in vivo*, we constructed a double-tagged strain (Fft3-TAP / Sfc4-Myc) and performed a co-immunoprecipitation assay. We observed an enrichment of Sfc4-Myc in the anti-TAP purified material from the double-tagged strain compared to the single-tagged strain (Fig. 6E), confirming that Fft3 and Sfc4 physically interact *in vivo*. Taken together, our data indicate that the Fft3 chromatin remodeler directly interacts with the TFIIIC transcription complex at Pol III transcribed loci.

### Fft3 affects peripheral positioning and nucleosome occupancy of tRNA genes

Although tRNA genes are dispersed throughout the fission yeast genome, most of them cluster into a few foci in close proximity to centromeres, facilitated by condensin [33,34]. We found condensin binding strongly to tRNA genes both in wild type cells and *fft3Δ* cells (S9 Fig), suggesting that clustering is unaffected the absence of Fft3. Since the tRNA genes cluster close to the centromeres and therefore the nuclear periphery [34], we asked whether their nuclear positioning changes in *fft3Δ* cells. While tRNA genes are enriched for Nup85 and Man1 interactions in wild type (Fig. 6F-G), we observed a reduction of Man1 association in *fft3Δ* cells (Fig. 6H). This suggests that tRNAs move away from the nuclear envelope in the absence of Fft3. Interestingly, this effect seems to be specific to tRNA genes, since the 5S rRNA genes stay enriched for Man1 association when Fft3 is deleted (S8B–D Fig).

We then wondered if there are further effects at tRNA genes in *fft3Δ* cells. Therefore, we examined expression levels of two tRNA classes, proline and alanine, by northern blot and RT-qPCR (S10A–C Fig). We observed an increase in transcription for proline, but not for alanine, suggesting there is no clear-cut effect of Fft3 on tRNA transcription. A clearer trend emerged when we looked at MNase sequencing data: like LTRs, tRNA genes show a pronounced decrease in nucleosome occupancy (Fig. 6I). Similarly, 5S rRNA genes also showed a decrease in nucleosome occupancy (S6E Fig). Together, these data indicate that Fft3 is involved in maintaining chromatin structure and intra-nuclear positioning of Pol III transcribed genes.

## Discussion

It has been established in different eukaryotic systems that the periphery of the cell nucleus harbors chromosomal domains with transcriptionally repressed chromatin. Insulators contribute to this three-dimensional nuclear organization by forming clusters to separate different chromatin domains [35].

**Fig 7:**
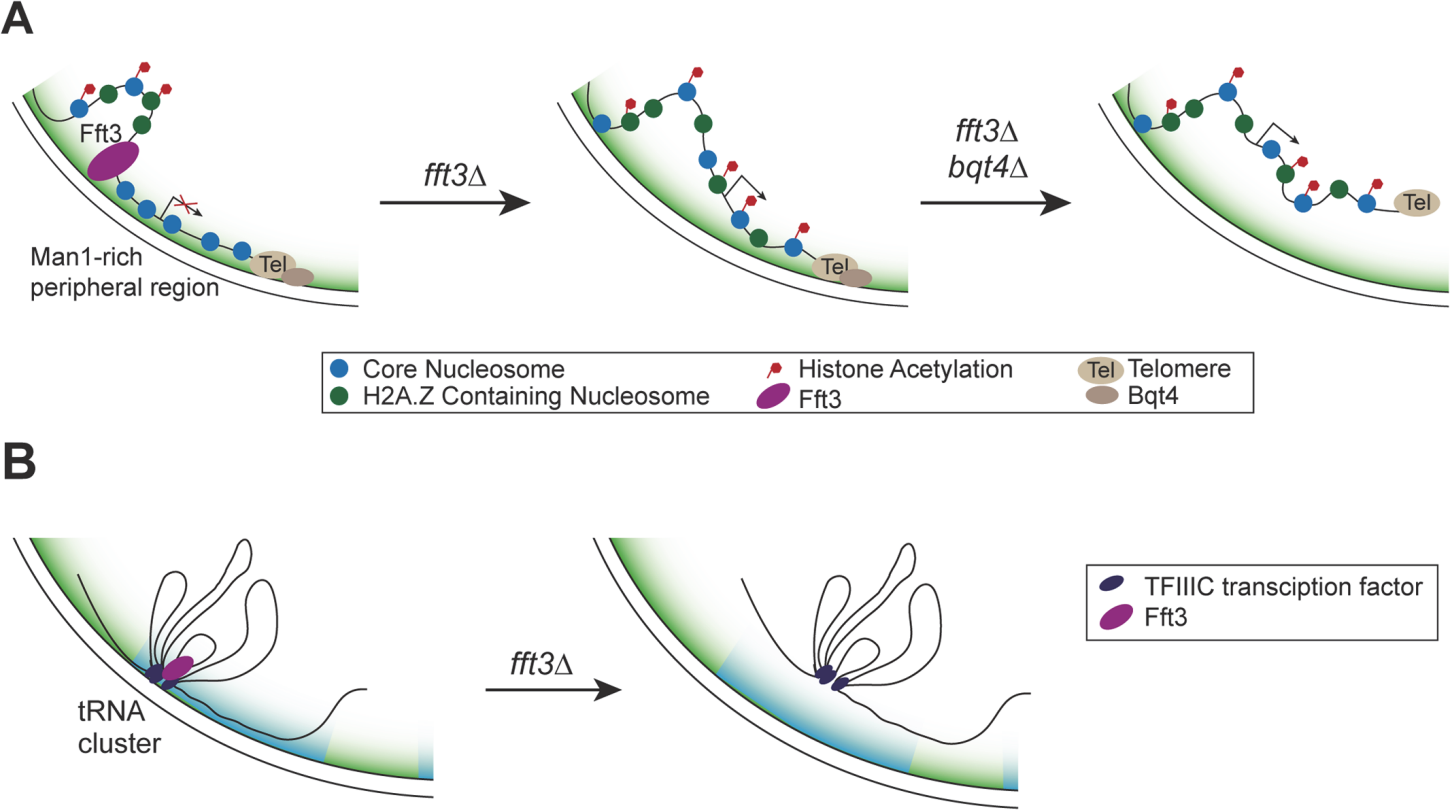
Proposed mechanism of how Fft3 regulates subtelomeric chromatin and tRNA positioning. **(A)** Fft3 maintains subtelomeric chromatin through binding and remodeling activity at boundary elements. In the absence of Fft3, active chromatin marks and transcription in the subtelomeric domain increase and the domain is released from the nuclear periphery. When Bqt4 is deleted in addition to Fft3, the entire telomere can move away from the nuclear envelope towards the interior. **(B)** tRNA clusters at the nuclear envelope dissociate from the periphery in *fft3Δ* cells and lose their transcriptional regulation.

Here we show that cells lacking the Fft3 remodeling enzyme or carrying a catalytically inactive version display drastic changes in chromatin marks and gene expression in the subtelomeric regions (Fig. 7A). Fft3 is required to ensure the association of these domains with the nuclear envelope through the LEM domain protein Man1. The budding yeast homolog of Man1, Heh1p, is involved in regulating silencing of rDNA repeats [36]. Also in budding yeast, subtelomeres are associated with the INM protein Src1p [37] and subtelomeric genes change in expression when the Fft3 homolog Fun30 is missing [38]. Our data highlights that Fft3—even though itself not enriched at the subtelomeres *per se*—affects gene expression in these domains by acting on their borders. We show that local chromatin structure at the borders is altered in *fft3Δ* cells. This agrees with observations on the action of other chromatin remodelers at insulators, such as incorporation of H3.3 by PBAP at border elements in fruit flies [39] and binding of ISWI to ArsI insulators in sea urchins [40].

The borders of subtelomeres in *S*. *pombe* coincide with retrotransposon-derived LTR elements. In mouse cells, LTR elements can function as insulators [41]. Furthermore, the well-characterized *gypsy* insulator in *Drosophila* is derived from the gypsy retrotransposon (reviewed in [42]). The Tf2 retrotransposons in fission yeast cluster within the nucleus to form Tf-bodies, and de-cluster in response to oxidative stress [43]. Our results show that Fft3 binds at or near LTR elements and affects their nucleosome occupancy and position in the nucleus. Taken together, these findings point to a conserved role for transposable elements in genome organization and insulation.

It has been suggested that insulator elements function by changing higher-order chromatin structure (reviewed in [44]). Insulators can interact with each other and tether the chromatin fiber to structural elements within the nucleus, e.g. nuclear pores. In this way, chromatin loops can form which separate euchromatin and heterochromatin domains. In *S*. *pombe*, *Tf2* transposons cluster into *Tf* bodies that play a role in genome organization [43]. Furthermore, the *Drosophila gypsy* insulators form specialized insulator bodies at the nuclear periphery, and transposon-derived MAR/SAR sequences can create chromatin loops [42,45]. In agreement with this, we found that *S*. *pombe* LTR elements localize close to the nuclear periphery and nuclear pores. It is possible that this anchoring of LTR elements helps to divide loops of chromatin with different properties, such as the subtelomeric chromatin and neighboring euchromatin.

In addition to LTR elements, Fft3 binds to tRNA genes and physically interacts with the Pol III transcription factor TFIIIC. These features may be conserved in the Fun30 remodeling family, since the *S*. *cerevisiae* homolog, Fun30, is enriched over tRNA genes and the human homolog, SMARCAD1, purifies together with the TFIIIC complex [16,17,21]. Based on our findings in *S*. *pombe* it is plausible that budding yeast and human Fun30 homologues also directly interact with TFIIIC. Like in *S*. *pombe*, tRNA genes function as insulators in other eukaryotes and play a role in genome organization by clustering at specific sites in the nucleus (reviewed in [46]). It is noteworthy that both the insulating function and clustering of tRNA genes in yeast depend on TFIIIC [46]. It is therefore likely that Fft3 is recruited to tRNA genes through its interaction with TFIIIC and maintains the chromatin state at these insulators. Fft3 does not appear to be involved in condensin recruitment to tRNA genes, suggesting that condensin-dependent clustering of tRNA genes may not be affected. However, our results indicate that Fft3 is required for their anchoring at the nuclear envelope (Fig. 7B).

At the nucleosome level, we observed a reduction in nucleosome occupancy at Fft3 targets when the remodeler was missing. A similar decrease has also been observed at genes regulated by Fun30 in budding yeast [38]. This suggests that Fft3 is required to incorporate these nucleosomes or to stabilize them after incorporation. The nucleosomes could then serve either as a platform for binding or as a barrier for other proteins. Alternatively, Fft3 remodeling could affect chromatin mobility. INO80, another Snf2 remodeling factor, facilitates chromatin movement inside the budding yeast nucleus by altering the stiffness of the chromatin fiber [47]. It is conceivable that Fft3 acts in a similar manner to allow for correct positioning of its targets within the nucleus.

Beyond yeasts, subtelomeres are important in several other eukaryotic systems. In parasites such as *Trypanosoma brucei* and *Plasmodium falciparum*, the subtelomeres habor genes encoding surface markers that can be varied through recombination [48] and contribute to antigen variation. In *T*. *brucei*, these loci move from the nuclear periphery towards the nuclear interior when activated during differentiation [49]. In human cells, the D4Z4 insulator is located in the subtelomere of chromosome 4q and maintains subtelomeric heterochromatin [50]. Deletion of D4Z4 causes a type of muscular dystrophy, Facio-Scapulo-Humeral Dystrophy (FSHD). Interestingly, insulator function of D4Z4 depends on CTCF and lamin A, and is involved in peripheral positioning of the telomere [51,52]. Taken together, these studies highlight the importance of links between subtelomeric chromatin states and nuclear positioning.

In this study, we provide an example how a chromatin remodeler affects both local chromatin structure and genome-wide nuclear organization. Further studies will be necessary to shed light on this interplay between nuclear architecture and transcriptional regulation that seems to have a major role genome function in eukaryotes.

## Materials and Methods

### Strain construction and cell culture

*S*. *pombe* cells were grown at 30°C and in YES medium unless stated otherwise. The *S*. *pombe* strains used in this study are listed in S1 Table. The point mutation in the ATPase domain of Fft3 was created using PCR with a primer containing the mutation. Genomic DNA was isolated from the Fft3-myc::hph strain (Hu1911) and part of the gene, the myc-tag, the hygromycin resistance gene and part of the 3’UTR were amplified by PCR using primers ATPase-F and ATPase-R (see S2 Table). The ATPase-F primer contained a mismatch introducing the K418R substitution (AAA to AGA). To improve the recombination efficiency, the fragment was elongated by a second PCR using ATPaseL-F and ATPase-R primers. The PCR product was then purified and electroporated into a wild type strain (Hu0029). Strains in which the construct had replaced the wild type *fft3+* gene through homologous recombination were selected by hygromycin resistance and confirmed by DNA sequencing.

### Chromatin immunoprecipitation

DNA was immunoprecipitated as described earlier [53] using 2μl of anti-H4K12Ac (ab1761, abcam), 2μl of anti-myc (9E10, Sigma), 1 μl of anti-GFP (ab290, abcam), or 3μl anti-RNA polymerase II CTD repeat (ab5408, abcam) antibodies per 100μl chromatin extracts.

For microarray hybridization, immunoprecipitated DNA was amplified to 5 μg DNA as described in [53], with the exception that in the second PCR, 5 mM dUTP was added to the reaction. Fragmentation, labeling and hybridization to the Affymetrix GeneChip *S*. *pombe* Tiling 1.0FR was performed by the Affymetrix core facility at Novum (http://apt.bea.ki.se) according to Affymetrix standard protocols.

All experiments were done as biological duplicates.

For real-time quantitative PCR, immunoprecipitated DNA was amplified in the presence of SYBR Green using Applied Biosystems 7500 real-time PCR machine. The primers used are listed in S2 Table.

### DamID

Genomic DNA was extracted as described previously [7], with an additional clean-up of genomic DNA using the DNeasy Blood and Tissue Kit (Qiagen). DamID experiments were done as described by [54]. The amplified material was cleaned using the Qiagen PCR purification kit and fragmented with DNAse. Fragmented DNA was labeled and hybridized to the Affymetrix GeneChip S. pombe Tiling 1.0FR using standard protocols by the Affymetrix core facility at Novum (http://apt.bea.ki.se).

All experiments were done as biological duplicates.

### RNA extraction

RNA was extracted using the hot phenol method as described in [55] and reverse transcribed using random hexamers and the SuperScript II Reverse Transcriptase kit (Invitrogen). Real-time quantitative PCR was performed in the presence of SYBR Green using an Applied Biosystems 7500 real-time PCR machine. Primers used are listed in S2 Table.

All experiments were done as biological duplicates.

### Fluorescence in-situ hybridization

Cells were grown to early log phase at 30°C in YES medium, fixed with adding paraformaldehyde to 1,75% for 40 min and processed for IF with mouse monoclonal anti-myc antibodies (9E10, Sigma, 4μg/ml). For FISH, cells were refixed with 3% paraformaldehyde for 30min at room temperature. Chromosomal DNA was denatured by successive 15 min incubation time in 2SSC, 2SSC 10% formamide, 2SSC 20% formamide and 2SCC 40% formamide at room temperature. Subtelomeric FISH probes were generated using the sequencing cosmid c186 (provided by the Wellcome Trust Sanger Institute, Hinxton, UK) as template. c186 spans 30176bp on chr1R (Range 5524764 to 5554939), from SPNCRNA.283 to SPAC186.09.

Images were taken using a Nikon A1+ laser scanning microscope with a 60x Lambda S oil-immersion objective (NA 1.4). Imaging was set up fulfilling the Nyquist criterion in xy and z, with a minimum zoom of 2. For each cell, z-stacks were acquired with 0.2μm spacing and subjected to a blind 3D deconvolution algorithm using the NIS Elements Advanced Research software version 4.12. After decapping (removing the top three and bottom three z-stacks) of each nucleus, distances from the center of each FISH spot to the nuclear periphery were measured. Measurements were then assigned to three concentric equal volume zones [56]. Significance tests were carried out using the Chi-square test.

### Live cell microscopy

Cells were grown over night at 30°C in PMG liquid medium. For mounting, 35 mm glass bottomed culture dishes (MatTek Corp) were coated with soy lectin (Sigma). A log phase cell suspension was added on top of the coated surface and cells were left to sediment for five minutes before pre-warmed PMG medium was added on top. During imaging, the culture dish was kept at 30°C inside the microscope incubator and cells were imaged for no longer than three hours.

Images were taken using an inverted Nikon A1R laser scanning microscope with a 60x Lambda S oil-immersion objective (NA 1.4). Imaging was set up fulfilling the Nyquist criterion in xy and z, with a minimum zoom of 2. For each nucleus, z-stacks were acquired with 0.125μm spacing in a 5 μm range around the center of the nucleus. Deconvolution of each stack was carried out using a 3D non-blind algorithm in NIS Elements (Nikon, High Content Analysis software version 4.20). After decapping (removing the top three and bottom three z-stacks of the nuclear volume) of each nucleus, distances from the center of each mCherry spot to the nuclear periphery were measured in the z-plane with the brightest signal. The radius of each nucleus was measured and used to normalize the distance measurement. Normalized distances were then assigned to one of three concentric equal volume zone [56]. Significance tests were carried out using a two-sided Chi-square test.

### Micrococcal nuclease digestion and sequencing

Mononucleosomal DNA fragments were digested and purified as described in [57]. Fragments were then amplified and labeled with the NEBNext ChIP-Seq Library Prep Master Mix Set for Illumina (NEB #E6240) followed by sequencing on an Illumina Miseq v.3. Paired-end reads were mapped to the *S*. *pombe* genome using Bowtie2[58] with standard parameters. DANPOS [59] was then used to remove clonal reads, normalize by quantile normalization and calculate nucleosome occupancy. All calculations and visualization were carried out using R. All experiments were done as biological duplicates. MNAse-seq data can be accessed at NCBI GEO under the accession number GSE58013.

### Protein extraction and western blot

Proteins were extracted using a FastPrep-24 machine (MP Biomedicals) and separated by SDS PAGE. Immunoblot analysis was carried out using anti-myc (9E10, Sigma) and anti-actin (ab8224, abcam) antibodies.

### Yeast-2-hybrid assay

The yeast-2-hybrid screen and data analysis were performed by Hybrigenics, Paris, France (www.hybrigenics.com). The full-length open-reading frame of the *fft3* gene (SPAC25A8.01c) was cloned into the pB27 vector as a C-terminal fusion to LexA. The construct was use as bait to screen a *S*. *pombe* cDNA library. 62.4 million interactions were tested.

### Co-immunoprecipitation

50 ml of log phase growing cells were pelleted and resuspended in 500μl lysis buffer (20 mM Tris pH7.5, 150mM NaCl, 3% glycerol, 0.05% Igepal, 0.5mM EDTA, 0.5 mM DTT and protease inhibitors) and lysed in a FastPrep machine (5x30sec at 6.5) with 1 volume of glass beads. The extract was centrifuged for 20 min, 14,000 rpm at 4°C, and the supernatant was incubated with 60μl 50% IgG-bead slurry for 40 min at 4°C. The beads were washed five times with lysis buffer. Bound proteins were eluted with 30μl NuPAGE LDS Sample Buffer (Invitrogen) at 70°C for 10 min and subjected to immunoblot analysis using anti-myc antibody (9E10, Sigma). The membrane was then stripped and incubated with anti-TAP antibody (CAB1001, Thermo Scientific)

### Northern blots

2.5ug of total RNA was electrophoresed in 10% polyacrylamide, 8M urea, 0.5x TBE gel and blotted onto Hybond Nx membrane (GE Healthcare) in 0.3x TBE using a semi dry blotter, followed by UV crosslinking. Oligonucleotide probes were ^32^P labelled using T4 PNK (USB Affymetrix) and hybridized in 5x Denhardt’s solution, 6xSSC, 10mM EDTA, 0.5% SDS, 0.1mg/ml salmon sperm DNA (Invitrogen) at respective temperatures for 16 hours. The blots were washed 3 times in 2xSSC 0.1% SDS and exposed to Phosphorimager screens (Fuji). Screens were scanned using Molecular Imager FX (BioRad). Band intensities were quantified using the Quantity One software (BioRad). Probe sequences are listed in S2 Table.

### Data analysis

All analysis was performed in R (http://www.r-project.org) using the Bioconductor (http://www.bioconductor.org) packages “affy”, “affxparser” and “preprocessCore” with standard parameters. CEL-files were imported and normalized as described in [7]. For visualization, all probes were used, while only probes with one match in the genome were used for calculations and significance tests.

To determine probe distributions for the subtelomeres, probes mapping to the following regions were used: 1–98950 bp and 5496300–5579133 bp on chromosome 1, 1–96400 bp and 4437300–4539804 bp on chromosome 2. For probe distributions across tRNA genes, probes were used which overlapped with sequence features marked “tRNA” in the GFF-annotation file downloaded from the Pombase website (ftp://ftp.sanger.ac.uk/pub/yeast/pombe/GFF/pombe_09052011.gff). For averaging of microarray scores across different genomic features, the “feature” column was used for all items with the following exceptions: features matching “LTRTF2”, “Tf2” (together with the feature category “protein_conding_gene”), “origin_of_replication” and “external_name = intron” were annotated as LTRs, Tf2s, replication origins and introns, respectively.

Microarray data published elsewhere was used as processed data when available or otherwise processed as described above. Boxplot were created in R using the “boxplot” function with standard parameters. Significance tests between data subsets (subtelomeric and tRNA probes vs. all other probes) were performed using a circular permutation as described in [7].

Microarray data can be accessed at NCBI GEO under the accession number GSE58013.

## Supporting Information

S1 Fig Verification of genome-wide data by qPCR. **(A-C)** Increase in enrichment of RNAP II, H2A.Z and H4K12Ac at subtelomeres was confirmed by qPCR. Data shown as fold difference in enrichment at SPBPB8B6.04c over actin, normalized to input. Error bars represent the standard deviation of duplicate experiments.
(PDF)

S2 Fig Loss of peripheral association was confirmed by qPCR. Data shown as fold difference in enrichment over Dam-only control. Error bars represent the standard deviation of duplicate experiments.
(PDF)

S3 Fig (A) FISH-Immunofluorescence examples for wt and *fft3Δ* cells. The nuclear envelope was stained with Nup61-myc-Cy3 (red) and the subtelomeric FISH probe was labeled with FITC-anti-DIG (green). Cells were scored as belonging to either of three equal volume zones depending on the distance between the nuclear envelope and the FISH signal. **(B)** Subtelomeres move towards the interior in *fft3Δ bqt4Δ cells*. Percentages of cells in each of the zones in different strains are shown, with number of cells measured in the legend. Significance testing was done using a two-sided Chi-square test.
(PDF)

S4 Fig Fft3 is enriched at LTR elements. **(A)** Nup85 is significantly enriched at LTR elements. Nup85-Dam enrichment over Dam-only control is shown as boxplot. **(B)** Nup85 levels decrease at LTRs in *fft3Δ* cells. Data shown as fold difference in enrichment at LTR elements over Dam-only control. Error bars represent the standard deviation of duplicate experiments. **(C)** The subtelomeric border element (SBE) LTRs on the left arm of chromosome 2 loose their association with the nuclear envelope in *fft3Δ* cells. Data shown as fold difference in enrichment at SBE LTRs over Dam-only control. Error bars represent the standard deviation of duplicate experiments. **(D)** Insertion of an LTR into the *ura4* gene is not sufficient to increase Fft3 interaction. Data from anti-myc ChIP qPCR of a strain with wild-type *ura4* and a strain with an LTR inserted into the *ura4* coding sequence (see schematic). Data is shown as enrichment over control locus (LTR close to SPBPB10D8.04c) with error bars representing the standard deviation of duplicate experiments.
(PDF)

S5 Fig Nucleosome occupancy changes over subtelomeric borders in *fft3Δ* cells. Nucleosome occupancy at subtelomeric borders is affected by Fft3. Map shows nucleosome occupancy profiles (top panel) and Fft3 enrichment (blue) over the border of **(A)** the right subtelomere on chromosome I, **(B)** the left subtelomere on chromosome II and **(C)** the right subtelomere on chromosome II. Genes are shown in light grey, LTRs in dark grey. Asterisks mark positions were nucleosome occupancy is reduced. The left subtelomere border on chromosome II contains four identical copies of the gene SPBPB10D8.04c and an LTR, so the profiles in **(B)** are shown as an average over all four copies.
(PDF)

S6 Fig Fft3-K418R-myc is recruited to the same locations as Fft3-myc, but mimics the *fft3Δ* phenotype. **(A)** The Fft3 ATPase mutant is recruited to the central core domain of centromere 1 (cnt1). Data from ChIP-qPCR of non-tagged wildtype, Fft3-myc and Fft3-K418R-myc is shown as fold difference in enrichment at Cnt1 over *dg*, normalized to input. Error bars represent the standard deviation of duplicate experiments. **(B)** The Fft3 ATPase mutant is recruited to valine tRNA genes. Data/error bars as in **(A)**. **(C)** The Fft3 ATPase mutant is recruited to proline tRNA genes. Data/error bars as in **(A)**. **(D)** *Fft3-K418R* cells show the same temperature sensitivity as *fft3Δ* cells. Cell suspensions were diluted into five different concentrations, spotted on YES plates and grown at the indicated temperatures for 3 days.
(PDF)

S7 Fig Fft3-K418R-myc shows increased active chromatin marks and decreased Man1-association at subtelomeres. **(A)** RNA Polymerase II levels are increased in *fft3Δ* and Fft3-K418R-myc. Data from ChIP-qPCR of non-tagged wild-type, *fft3Δ* and Fft3-K418R-myc is shown as percent of input. Error bars represent the standard deviation of duplicate experiments. **(B)** H3K9Ac levels are increased in *fft3Δ* and Fft3-K418R-myc. Data/error bars as in **(A),** except that data was normalized to H3 occupancy. **(C)** H2A.Z levels are increased in *fft3Δ* and Fft3-K418R-myc. Data/error bars as in **(A)**. **(D)** Man1-interaction is reduced in *Fft3-K418R* cells compared to wild-type. Data from DamID qPCR is shown as Man1-enrichment over Dam-only control. Error bars represent the standard deviation of duplicate experiments.
(PDF)

S8 Fig 5S rRNA genes are peripheral and bound by Fft3. **(A)** Fft3 binds to various genomic elements, e.g. LTRs, tRNA genes and snRNA genes. Boxplots show averages of Fft3-myc enrichment over anti-myc control for each class. **(B)** Fft3 is enriched over 5S rRNA genes. Fft3-enrichment over wildtype anti-myc control is shown as boxplot. Fft3-myc is ChIP-chip data from [14]. **(C)** Nup85 is enriched at 5S rRNA genes in wildtype *S*. *pombe*. Nup85-Dam enrichment over Dam-only control is shown as boxplot. Nup85-Dam data is from [24]. **(D)** The INM protein Man1 is enriched at 5S rRNA genes in wildtype *S*. *pombe*. Man1-Dam enrichment over Dam-only control is shown as boxplot. **(E)** Fft3 is not required for 5S rRNA peripheral association. Man1-Dam enrichment over Dam-only control in *fft3Δ* cells is shown as boxplot. **(F)** Nucleosome occupancy at 5S rRNA genes is reduced in *fft3Δ* cells. The average number of reads mapping to each rRNA is shown for wild type and *fft3Δ*. P-value was obtained using paired, two-sided Mann-Whitney U test.
(PDF)

S9 Fig Fft3 does not affect condensin binding to tRNA genes. Enrichment of the condensin subunit Cnd2 over anti-GFP control is shown as boxplot. Scores for probes mapping to 129 of the total 170 tRNA genes are shown in dark grey, scores for all other probes shown in light grey.
(PDF)

S10 Fig Fft3 affects tRNA gene transcription in some, but not all, tRNA classes. **(A)** Levels of Proline tRNAs, but not Alanine tRNAs are increased in *fft3Δ* and *fft3-K418R* cells, compared to wildtype. Northern blots of total RNA were probed with ^32^P-labelled oligonucleotide probes, using snoRNA33 as a control for equal loading. **(B)** Quantification of the tRNA levels in (A). The log2-ratio of *fft3Δ* / *wildtype* is shown in dark grey, the log2-ratio of *fft3-K418R-myc* / *fft3-myc* is shown in light grey. Error bars represent the standard deviation of duplicate experiments. **(C)** Expression changes of Proline and Alanine tRNAs verified by qPCR. Total RNA was extracted and reverse transcribed into cDNA. The expression levels of two tRNA classes were measured with RT-qPCR. Error bars represent the standard deviation of duplicate experiments.
(PDF)

S1 Table List of strains used in this study. (PDF)

S2 Table List of primers used in this study. (PDF)
