# Supplementary figures and images for: The Fun30 Chromatin Remodeler Fft3 Controls Nuclear Organization and Chromatin Structure of Insulators and Subtelomeres in Fission Yeast
Source: PLoS Genet. 2015 Mar 23;11(3):e1005101. doi: 10.1371/journal.pgen.1005101 (PMC4370569; doi:10.1371/journal.pgen.1005101)

Figure S1

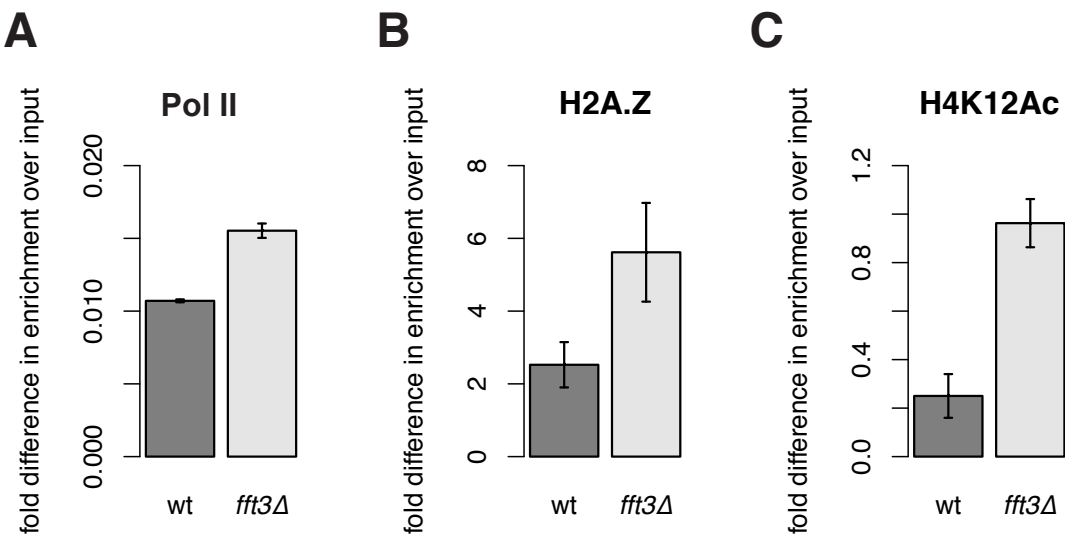

Supplement: S1 Fig — (A-C) Increase in enrichment of RNAP II, H2A.Z and H4K12Ac at subtelomeres was confirmed by qPCR. Data shown as fold difference in enrichment at SPBPB8B6.04c over actin, normalized to input. Error bars represent the standard deviation of duplicate experiments. (PDF) [file pgen.1005101.s001.pdf]

**Figure S2**

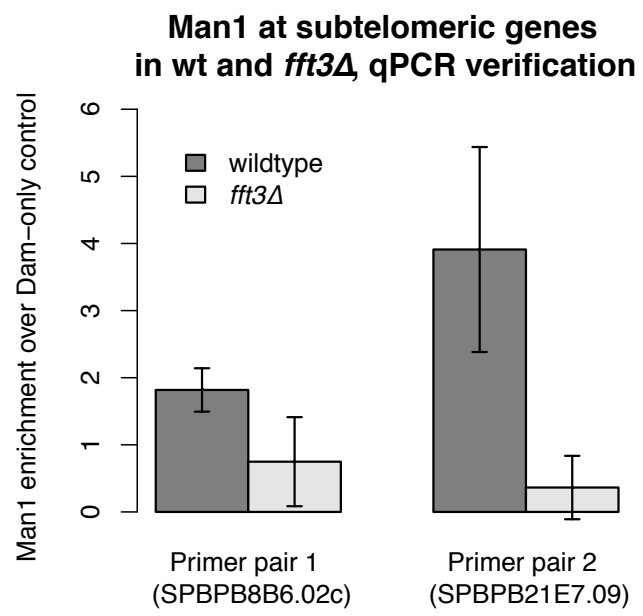

Supplement: S2 Fig — Data shown as fold difference in enrichment over Dam-only control. Error bars represent the standard deviation of duplicate experiments. (PDF) [file pgen.1005101.s002.pdf]

Figure S3

A

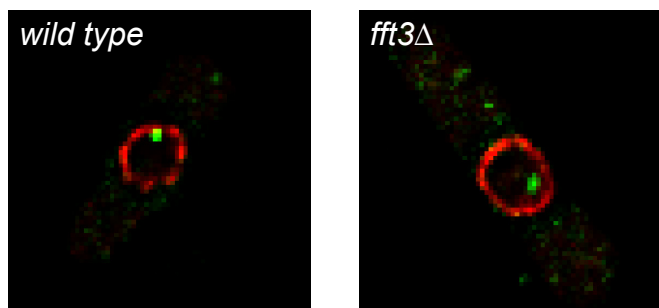

B

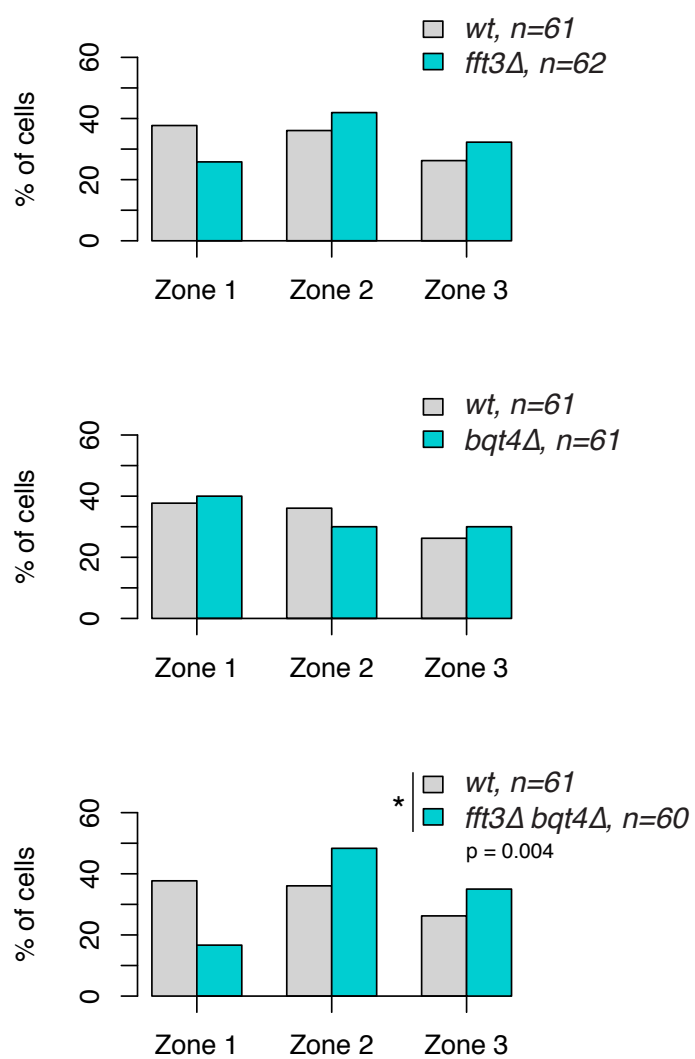

Supplement: S3 Fig — The nuclear envelope was stained with Nup61-myc-Cy3 (red) and the subtelomeric FISH probe was labeled with FITC-anti-DIG (green). Cells were scored as belonging to either of three equal volume zones depending on the distance between the nuclear envelope and the FISH signal. (B) Subtelomeres move towards the interior in fft3Δ bqt4Δ cells. Percentages of cells in each of the zones in different strains are shown, with number of cells measured in the legend. Significance testing was done using a two-sided Chi-square test. (PDF) [file pgen.1005101.s003.pdf]

Figure S4

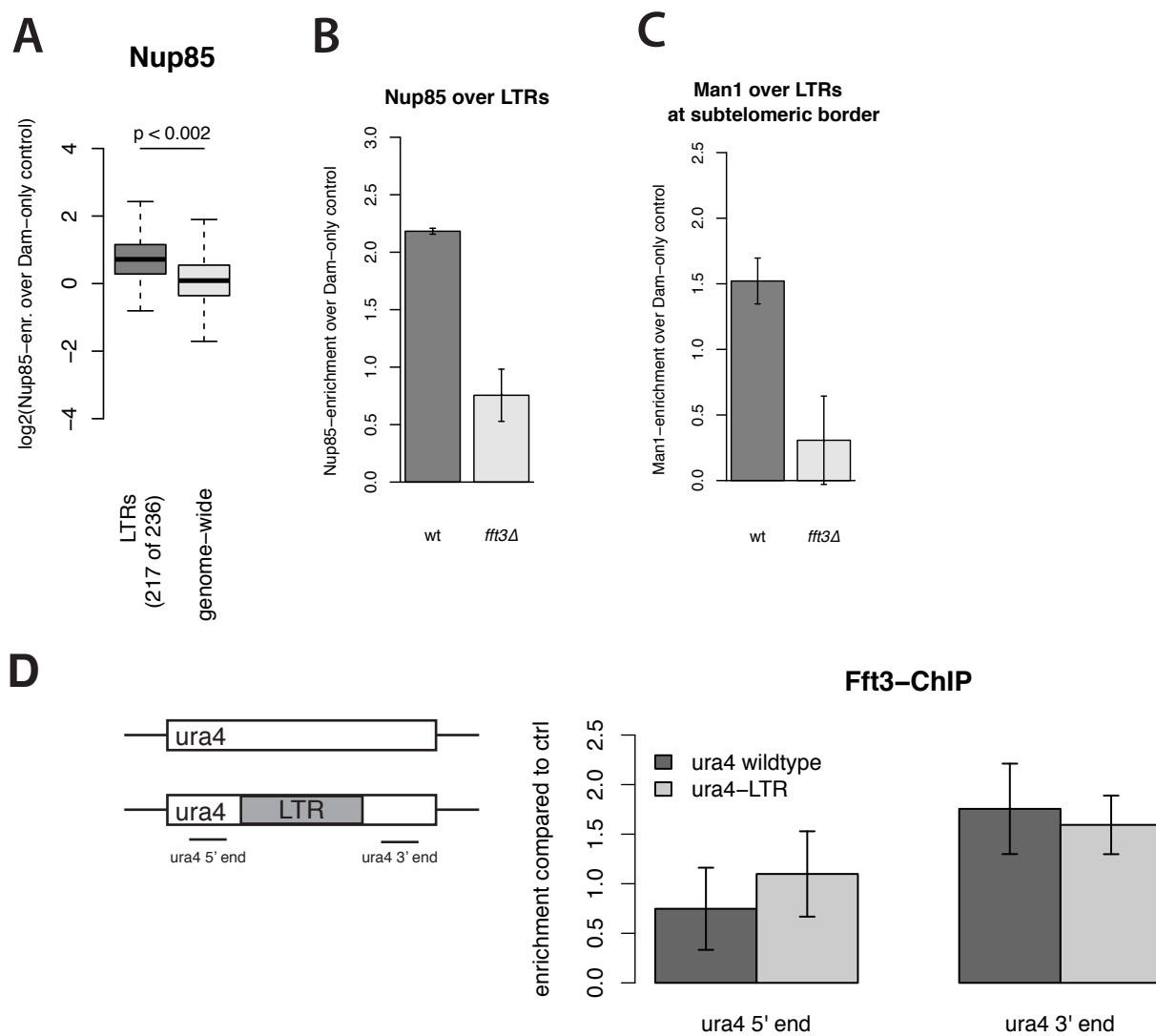

Supplement: S4 Fig — (A) Nup85 is significantly enriched at LTR elements. Nup85-Dam enrichment over Dam-only control is shown as boxplot. (B) Nup85 levels decrease at LTRs in fft3Δ cells. Data shown as fold difference in enrichment at LTR elements over Dam-only control. Error bars represent the standard deviation of duplicate experiments. (C) The subtelomeric border element (SBE) LTRs on the left arm of chromosome 2 loose their association with the nuclear envelope in fft3Δ cells. Data shown as fold difference in enrichment at SBE LTRs over Dam-only control. Error bars represent the standard deviation of duplicate experiments. (D) Insertion of an LTR into the ura4 gene is not sufficient to increase Fft3 interaction. Data from anti-myc ChIP qPCR of a strain with wild-type ura4 and a strain with an LTR inserted into the ura4 coding sequence (see schematic). Data is shown as enrichment over control locus (LTR close to SPBPB10D8.04c) with error bars representing the standard deviation of duplicate experiments. (PDF) [file pgen.1005101.s004.pdf]

Figure S5

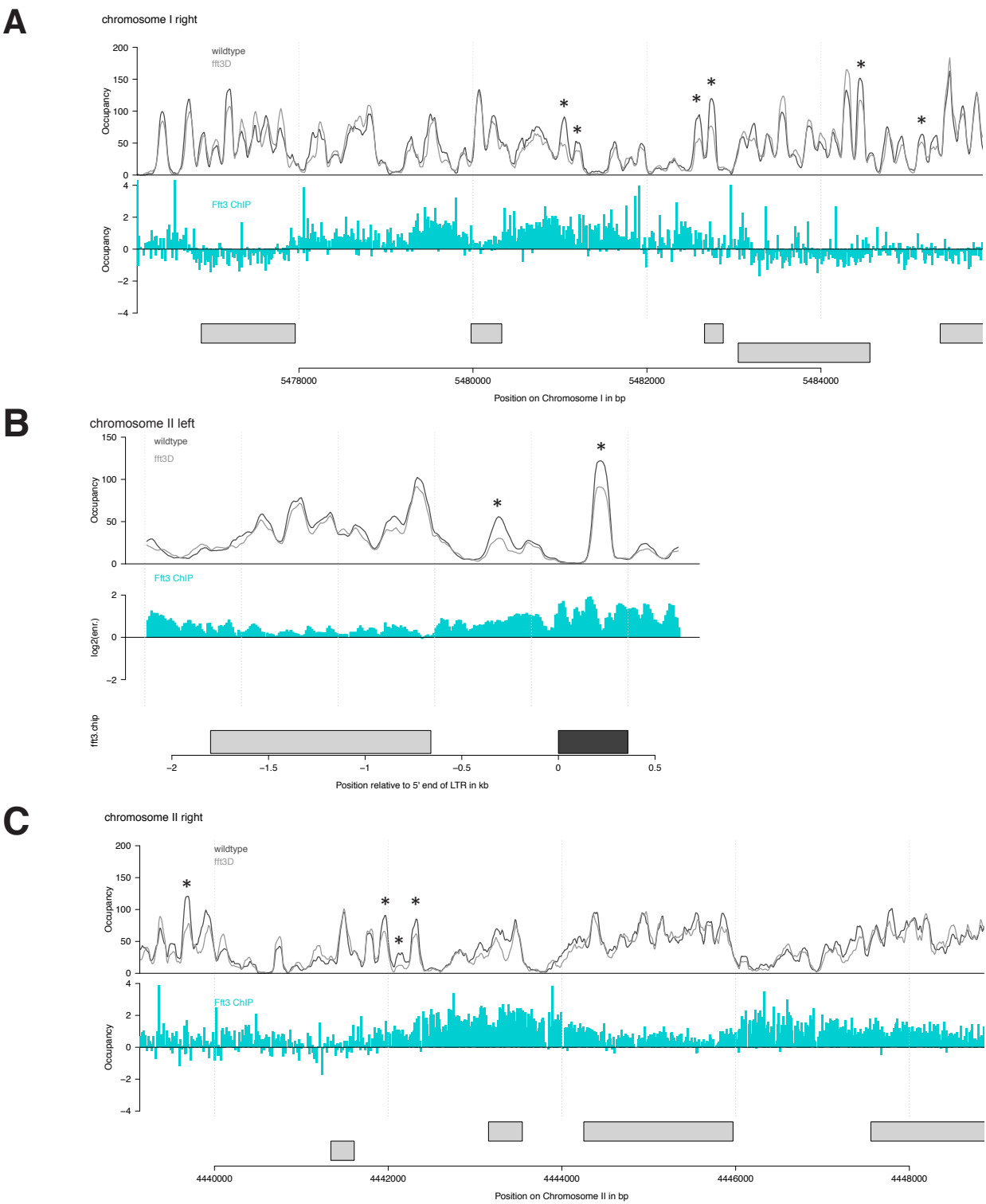

Supplement: S5 Fig — Nucleosome occupancy at subtelomeric borders is affected by Fft3. Map shows nucleosome occupancy profiles (top panel) and Fft3 enrichment (blue) over the border of (A) the right subtelomere on chromosome I, (B) the left subtelomere on chromosome II and (C) the right subtelomere on chromosome II. Genes are shown in light grey, LTRs in dark grey. Asterisks mark positions were nucleosome occupancy is reduced. The left subtelomere border on chromosome II contains four identical copies of the gene SPBPB10D8.04c and an LTR, so the profiles in (B) are shown as an average over all four copies. (PDF) [file pgen.1005101.s005.pdf]

**Figure S6**

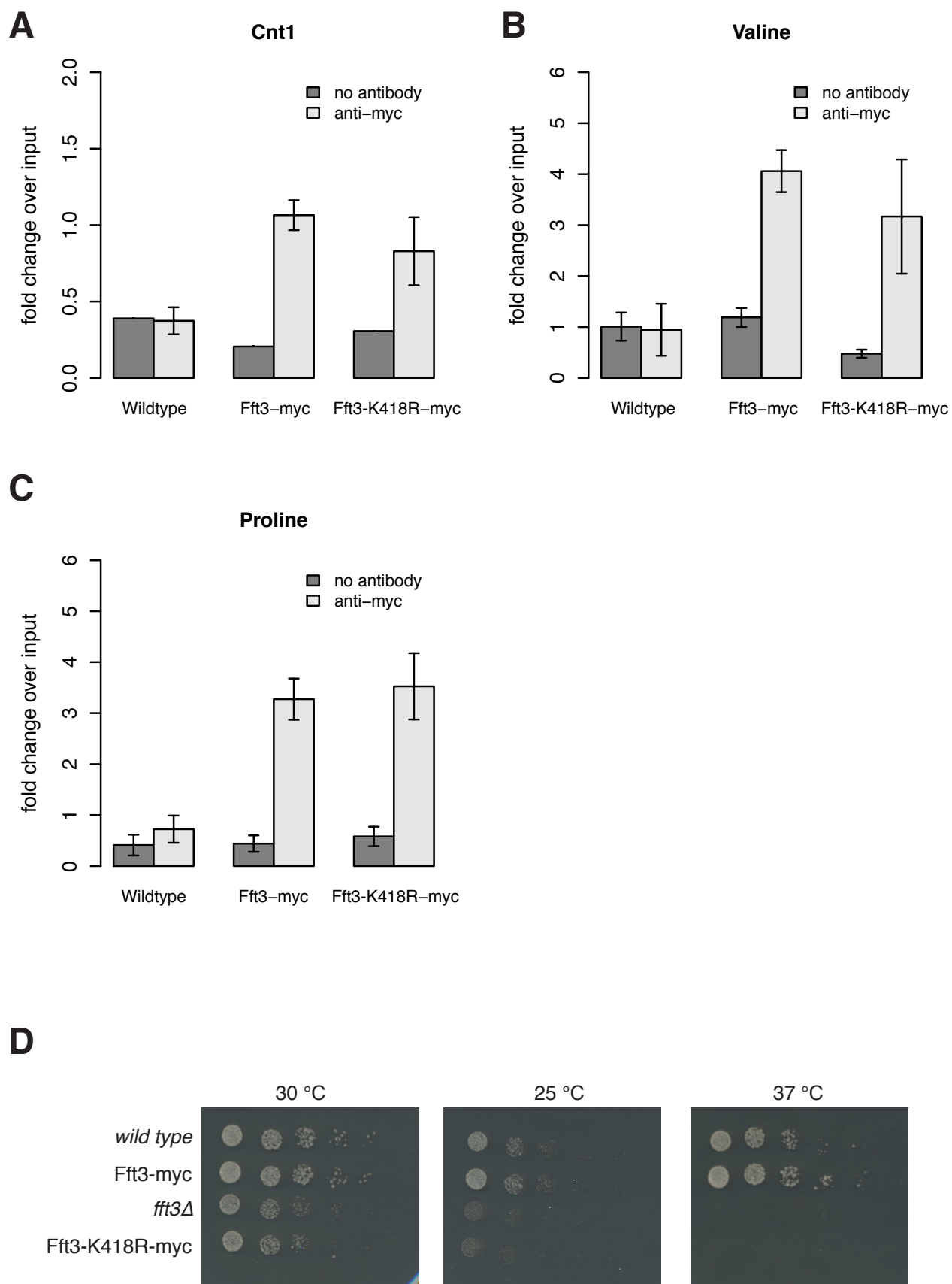

Supplement: S6 Fig — (A) The Fft3 ATPase mutant is recruited to the central core domain of centromere 1 (cnt1). Data from ChIP-qPCR of non-tagged wildtype, Fft3-myc and Fft3-K418R-myc is shown as fold difference in enrichment at Cnt1 over dg, normalized to input. Error bars represent the standard deviation of duplicate experiments. (B) The Fft3 ATPase mutant is recruited to valine tRNA genes. Data/error bars as in (A). (C) The Fft3 ATPase mutant is recruited to proline tRNA genes. Data/error bars as in (A). (D) Fft3-K418R cells show the same temperature sensitivity as fft3Δ cells. Cell suspensions were diluted into five different concentrations, spotted on YES plates and grown at the indicated temperatures for 3 days. (PDF) [file pgen.1005101.s006.pdf]

**Figure S7**

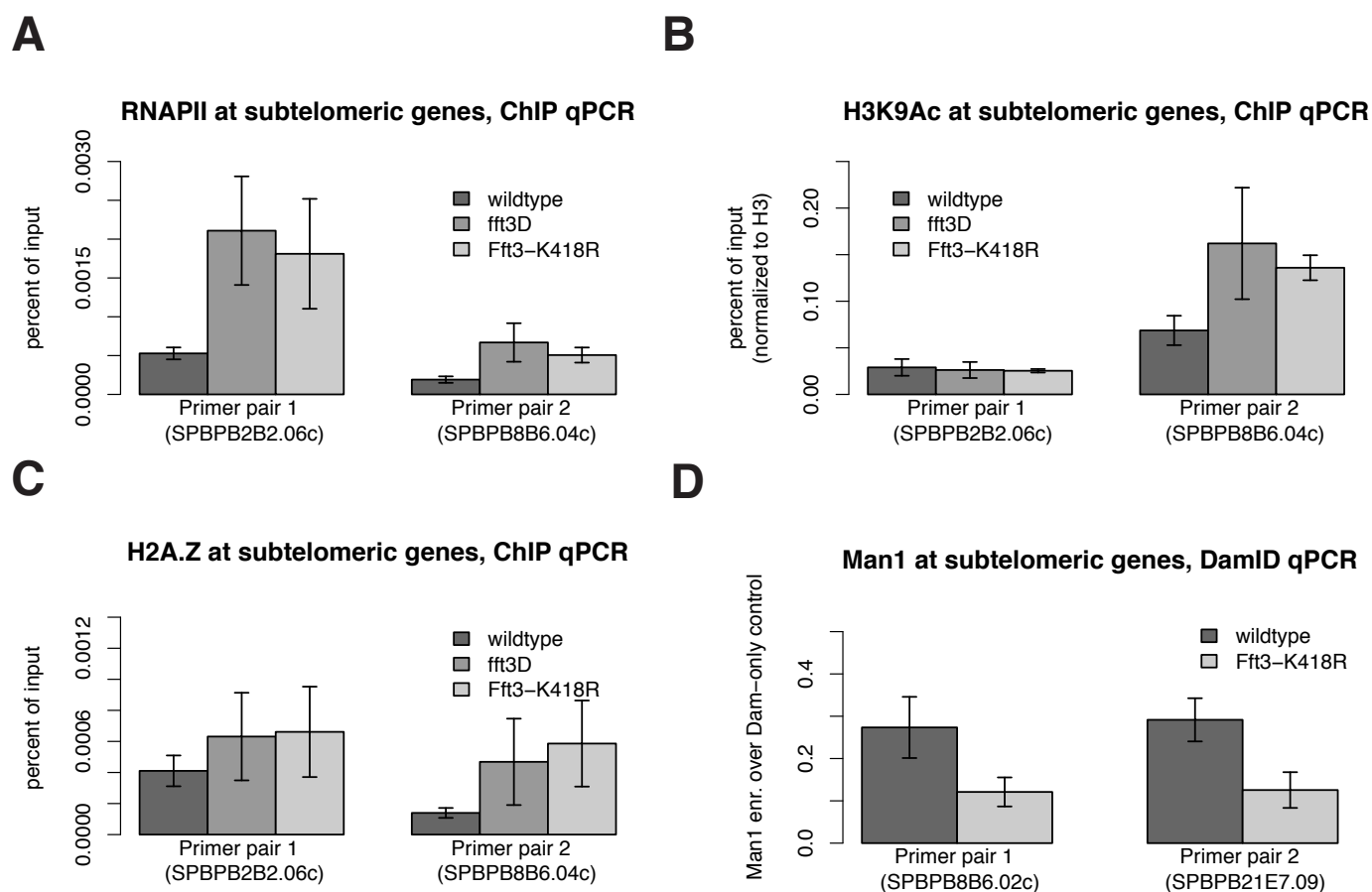

Supplement: S7 Fig — (A) RNA Polymerase II levels are increased in fft3Δ and Fft3-K418R-myc. Data from ChIP-qPCR of non-tagged wild-type, fft3Δ and Fft3-K418R-myc is shown as percent of input. Error bars represent the standard deviation of duplicate experiments. (B) H3K9Ac levels are increased in fft3Δ and Fft3-K418R-myc. Data/error bars as in (A), except that data was normalized to H3 occupancy. (C) H2A.Z levels are increased in fft3Δ and Fft3-K418R-myc. Data/error bars as in (A). (D) Man1-interaction is reduced in Fft3-K418R cells compared to wild-type. Data from DamID qPCR is shown as Man1-enrichment over Dam-only control. Error bars represent the standard deviation of duplicate experiments. (PDF) [file pgen.1005101.s007.pdf]

**Figure S8**

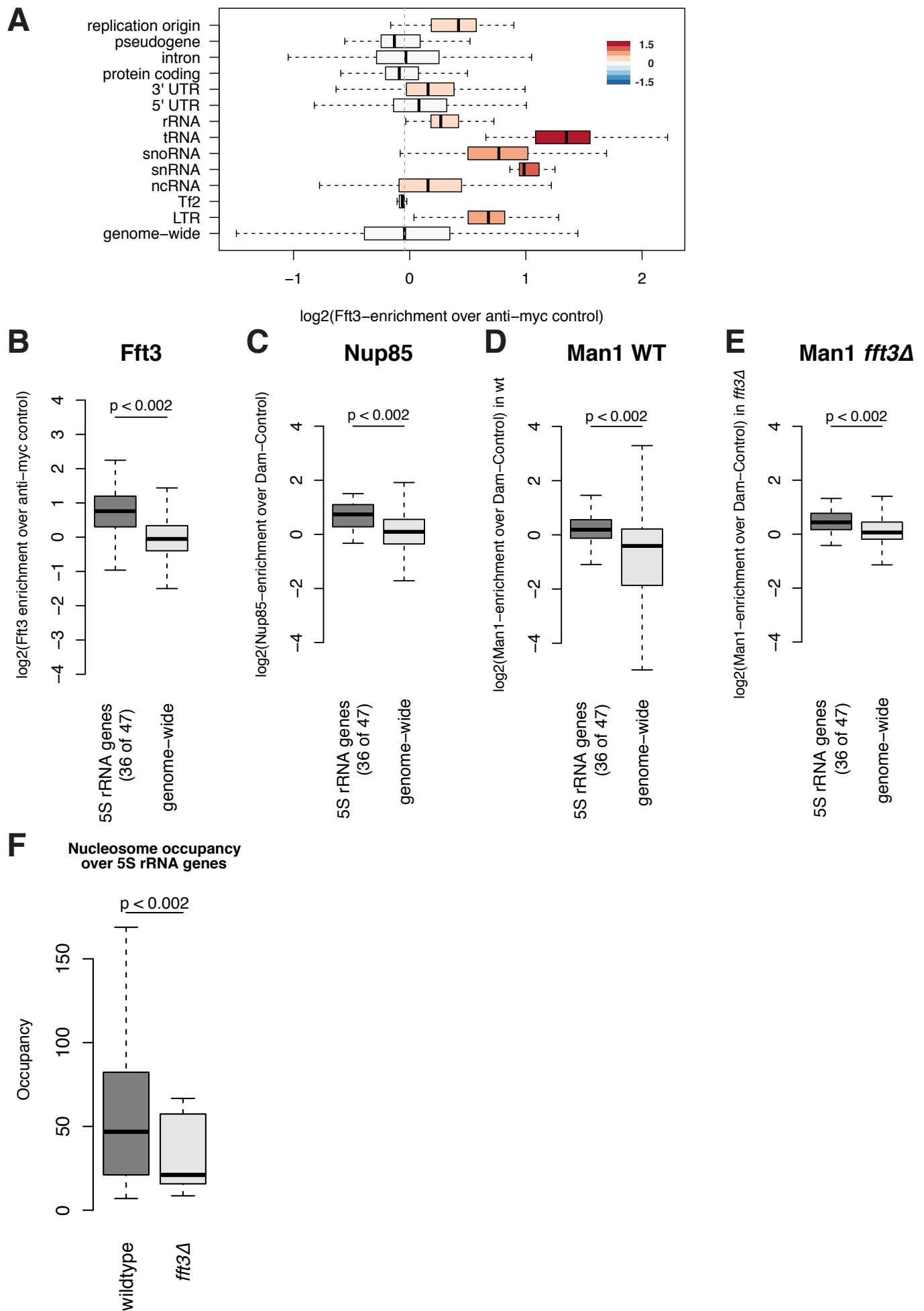

Supplement: S8 Fig — (A) Fft3 binds to various genomic elements, e.g. LTRs, tRNA genes and snRNA genes. Boxplots show averages of Fft3-myc enrichment over anti-myc control for each class. (B) Fft3 is enriched over 5S rRNA genes. Fft3-enrichment over wildtype anti-myc control is shown as boxplot. Fft3-myc is ChIP-chip data from [14]. (C) Nup85 is enriched at 5S rRNA genes in wildtype S. pombe. Nup85-Dam enrichment over Dam-only control is shown as boxplot. Nup85-Dam data is from [24]. (D) The INM protein Man1 is enriched at 5S rRNA genes in wildtype S. pombe. Man1-Dam enrichment over Dam-only control is shown as boxplot. (E) Fft3 is not required for 5S rRNA peripheral association. Man1-Dam enrichment over Dam-only control in fft3Δ cells is shown as boxplot. (F) Nucleosome occupancy at 5S rRNA genes is reduced in fft3Δ cells. The average number of reads mapping to each rRNA is shown for wild type and fft3Δ. P-value was obtained using paired, two-sided Mann-Whitney U test. (PDF) [file pgen.1005101.s008.pdf]

Figure S9

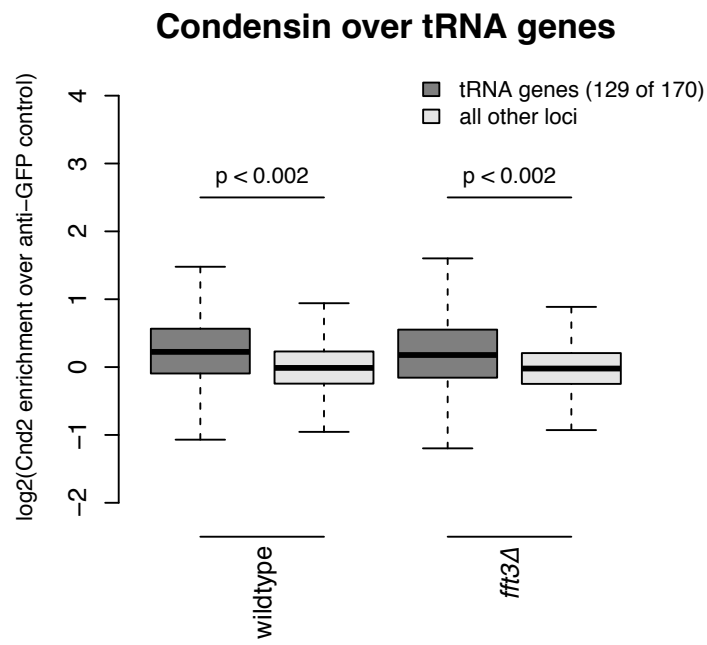

Supplement: S9 Fig — Enrichment of the condensin subunit Cnd2 over anti-GFP control is shown as boxplot. Scores for probes mapping to 129 of the total 170 tRNA genes are shown in dark grey, scores for all other probes shown in light grey. (PDF) [file pgen.1005101.s009.pdf]

Figure S10

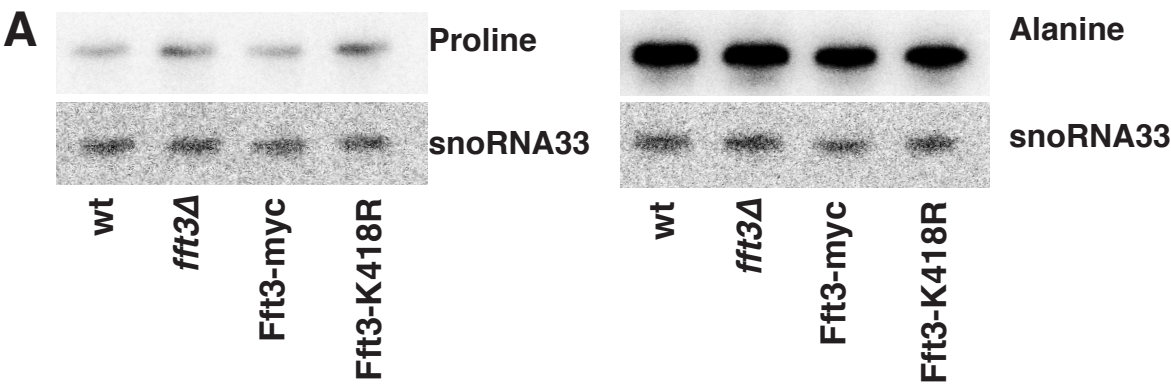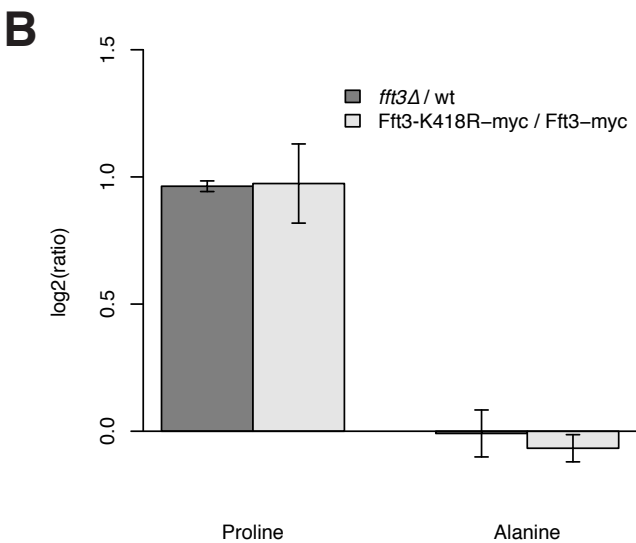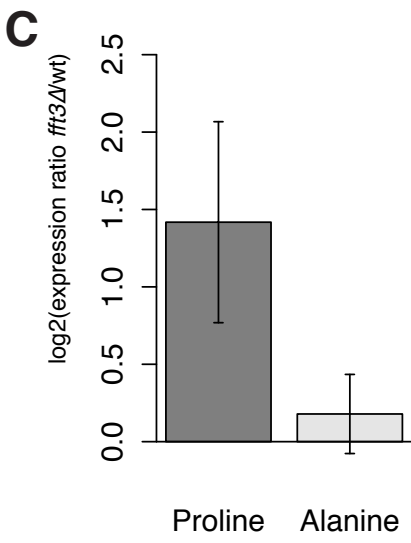

Supplement: S10 Fig — (A) Levels of Proline tRNAs, but not Alanine tRNAs are increased in fft3Δ and fft3-K418R cells, compared to wildtype. Northern blots of total RNA were probed with 32P-labelled oligonucleotide probes, using snoRNA33 as a control for equal loading. (B) Quantification of the tRNA levels in (A). The log2-ratio of fft3Δ / wildtype is shown in dark grey, the log2-ratio of fft3-K418R-myc / fft3-myc is shown in light grey. Error bars represent the standard deviation of duplicate experiments. (C) Expression changes of Proline and Alanine tRNAs verified by qPCR. Total RNA was extracted and reverse transcribed into cDNA. The expression levels of two tRNA classes were measured with RT-qPCR. Error bars represent the standard deviation of duplicate experiments. (PDF) [file pgen.1005101.s010.pdf]
