# Supplementary material for: The Fun30 Chromatin Remodeler Fft3 Controls Nuclear Organization and Chromatin Structure of Insulators and Subtelomeres in Fission Yeast
Source: PLoS Genet. 2015 Mar 23;11(3):e1005101. doi: 10.1371/journal.pgen.1005101 (PMC4370569; doi:10.1371/journal.pgen.1005101)
Supplement: S1 Table — (PDF) [file pgen.1005101.s011.pdf]

Table S1: List of strains used in this study

Strain	Genotype	Reference
Hu29	*h- ade6-M210 leu1-32 ura4-D18*	[1]
Hu1867	*h+ fft3::hph ade6-M210 uraDS/E leu1-32*	[1]
Hu1824	*h- pht1-myc::KanMx6 leu1-32 ade6-M210 ura4-D18*	[3]
Hu1993	*h- fft3::hph pht1-myc::KanMx6 leu1-32 ade6-M210 ura4-*	[3]
Hu2130	*h- Man1-7myc-Dam:NAT (nmt41 promoter)*	[4]
Hu2078	*h- 7myc-Dam-NLS:NATr (nmt41 promoter)*	[4]
Hu2193	*fft3::hph Man1-7myc-Dam:Nat (nmt41 promoter)*	This study
Hu2206	*fft3::hph 7myc-Dam-NLS:NATr (nmt41 promoter)*	This study
Hu2417	*h- fft3-K418R-myc::hph ade6-M210, ura4-D18, leu1-32*	This study
Hu1911	*h+ fft3-myc::hph leu1-32 ade6-210 ura4-D18*	This study
Hu1271	*h+ fft3-TAP Kan-MX6 leu1-32 ade6-210 ura4-D18*	This study
Hu2330	*h- sfc4-myc::kan ade6-M210 leu1-32 ura4-D18*	This study
Hu2343	*sfc4-myc::kan fft3-TAP::hph ade6-M210 leu1-32 ura4-D18*	This study
Hu2192	*h+ nup61::kan, ade6-M216, ura4-D18, leu1-32*	Bioneer Deletion Collection
Hu2173	*h+ man1::kan, ade6-M216, ura4-D18, leu1-32*	Bioneer Deletion Collection
Hu2413	*cnd2-GFP::NatR*	This study
Hu2414	*cnd2-GFP::NatR fft3D::HygR*	This study
Hu802	*h- clr6-1 (ts)*	[5]
Hu2108	*clr6-1 (ts) fft3::hph ade6-M210? uraDS/E? leu1-32?*	This study
Hu369	*h- clr3::kanMX ade6-M210, ura4-D18, leu1-32*	[5]
Hu2102	*fft3::hph clr3::KanMX adeM210 leu1-32 ura4-*	This study
Hu1026	*h- hos2::LEU2 leu1-32*	[5]
Hu2117	*fft3::hph hos2::leu2 leu1-32 ade6M210? uraDS/E?*	This study
Hu2424	*h+ otr1R(SphI)::ura4+ ura4-DS/E ade6-M210 leu1Δ::nmt1(81x)-dam-myc-nup85-kan*	[2]
Hu2422	*h+ otr1R(SphI)::ura4+ ura4-DS/E ade6-M210 leu1Δ::nmt1(81x)-dam-myc-kan*	[2]
Hu2438	*fft3::HygR leu1D::nmt1(81x)-dam-myc-nup85-KanR*	This study
Hu2434	*fft3::HygR leu1D::nmt1(81x)-dam-myc-KanR*	This study
Hu2582	*h90 taz1-mCherry::kanR hus5+::ccr1N-GFP::LEU2 leu1-32 lys1-131 ura4-D18 ade6-m210*	[6]
Hu2583	*h90 taz1-mCherry::kanR hus5+::ccr1N-GFP::LEU2 leu1-32 bqt4D::ura4+ lys1-131 ura4-D18 ade6-m210*	[6]
Hu2575	*h90 taz1-mCherry::kanR hus5+::ccr1N-GFP::LEU2 leu1-32 fft3D::HygR lys1-131 ura4-D18 ade6-m210*	This study
Hu2595	*h90 taz1-mCherry::kanR hus5+::ccr1N-GFP::LEU2 leu1-32 bqt4D::ura4+ fft3D::HygR lys1-131 ura4-D18 ade6-m210*	This study
Hu2241	*h- Nup61-myc::Kan ade6-M210 ura4-D18 leu1-32*	This study
Hu2256	*h- Nup61-myc::Kan fft3D::Hyg ade6-M210 ura4-D18orDS/E leu1-32*	This study
Hu2530	*h- Nup61-myc::Kan man1D::Kan ade6-M21X ura4-D18 leu1-32*	This study
Hu2566	*h- Nup61-myc::Kan bqt4D::ura4+ ade6-M21X ura4-D18 leu+*	This study
Hu2568	*h- Nup61-myc::Kan bqt4D::ura4+ fft3D::Hyg ade6-M21X ura4-D18orDS/E leu1-32*	This study
Hu2446	*fft3-K418R-myc::Hyg 7myc-Dam-NLS:NAT (nmt41 promoter)*	This study
Hu2448	*fft3-K418R-myc::Hyg Man1-7myc-Dam:NAT (nmt41 promoter)*	This study
Hu2771	*h- pht1-GFP::NAT leu1-32 ura4-*	[7]
Hu2791	*pht1-GFP::NAT fft3::HygR*	This study
Hu2797	*pht1-GFP::NAT fft3-K418R-myc::HygR*	This study
Hu2779	*fft3-myg::HygR ura4+*	This study
Hu2803	*fft3-myg::HygR ura4::ura4-LTR*	This study

References in Supplemental Material

1. Strålfors A, Walfridsson J, Bhuiyan H, Ekwall K (2011) The FUN30 chromatin remodeler, Fft3, protects centromeric and subtelomeric domains from euchromatin formation. PLoS Genet 7: e1001334. doi:10.1371/journal.pgen.1001334.

2. Woolcock KJ, Stunnenberg R, Gaidatzis D, Hotz H-R, Emmerth S, et al. (2012) RNAi keeps Atf1-bound stress response genes in check at nuclear pores. Genes & Development 26: 683–692. doi:10.1101/gad.186866.112.

3. Buchanan L, Durand-Dubief M, Roguev A, Sakalar C, Wilhelm B, et al. (2009) The Schizosaccharomyces pombe JmjC-protein, Msc1, prevents H2A.Z localization in centromeric and subtelomeric chromatin domains. PLoS Genet 5: e1000726. doi:10.1371/journal.pgen.1000726.

4. Steglich B, Filion GJ, van Steensel B, Ekwall K (2012) The inner nuclear membrane proteins Man1 and Ima1 link to two different types of chromatin at the nuclear periphery in S. pombe. Nucleus 3: 77–87.

5. Wirén M, Silverstein RA, Sinha I, Walfridsson J, Lee H-M, et al. (2005) Genome wide analysis of nucleosome density histone acetylation and HDAC function in fission yeast. The EMBO Journal 24: 2906–2918.

6. Chikashige Y, Yamane M, Okamasa K, Tsutsumi C, Kojidani T, et al. (2009) Membrane proteins Bqt3 and -4 anchor telomeres to the nuclear envelope to ensure chromosomal bouquet formation. The Journal of Cell Biology 187: 413–427. doi:10.1083/jcb.200902122.

7. Ogiyama Y, Ohno Y, Kubota Y, Ishii K (2013) Epigenetically induced paucity of histone H2A.Z stabilizes fission-yeast ectopic centromeres. Nature Structural & Molecular Biology 20: 1397–1406. doi:10.1038/nsmb.2697.
