# Supplementary material for: The Fun30 Chromatin Remodeler Fft3 Controls Nuclear Organization and Chromatin Structure of Insulators and Subtelomeres in Fission Yeast
Source: PLoS Genet. 2015 Mar 23;11(3):e1005101. doi: 10.1371/journal.pgen.1005101 (PMC4370569; doi:10.1371/journal.pgen.1005101)
Supplement: S2 Table — (PDF) [file pgen.1005101.s012.pdf]

Table S2: List of primers used in this study

Primer name	sequence
ATPase-F	TGATGAAATGGGGCTCGGTAGAACTTGTCAAACCATCGCTT
ATPase-R	TTTTCACGACGCGTTTTTGGCGTGGTATATAAATGG
ATPaseL-F	GATCATTGGGATTAATTGGCTTTACCTTTTGTACGAGCTCAAACTGGCT GGTATCCTGGCTGATGAAATGGGGCT
Dg1-F	AAT TGT GGT GGT GTG GTA ATA C
Dg1-R	GGG TTC ATC GTT TCC ATT CAG
LTR_SEB-F	ACG TAG TGC ATC ATA GCG TAG TGT AGT
LTR_SEB-R	ACT CGC AGC AAA AAT CAT GGT
Act1-F	TGA GGA GCA CCC TTG CTT GT
Act1-R	TCT TCT CAC GGT TGG ATT TGG
SPBPB2B2.06c-F	CACTCACGGTTGGCTTAATGG
SPBPB2B2.06c-R	TTCGCCAAAGTCGGCTTTAT
SPBPB8B6.04c-F	GTGTTGTCAAAAAGACCAGTTCGT
SPBPB8B6.04c-R	CACCTTGACTTTTCGTTTCCTACA
SPBPB8B6.02c-F	CTCTGGTGCTTTCAAGCGAAT
SPBPB8B6.02c-R	TGGCAAGGCCGAAAATCAT
SPBPB21E7.09-F	TGGGAGGAACTATCGCTGGAT
SPBPB21E7.09-R	CCAGCGGCATAGTCAACTGTT
Cen1-F	CAGACAATCGCATGGTACTATC
Cen1-R	AGGTGAAGCGTAAGTGAGTG
tRNA_Pro_l	GGCCGTTTGGTCTAGTGGTA
tRNA_Pro_r	GCTGTTGTGGGAATCGAAC
tRNA_Val_l	GGTCGTGTGGTTTAGATGGTT
tRNA_Val_r	ATCGTGCCAAGACTCGAACT
SPBPB2B2.01-F	GGAGCTATTGGTACCGGTGTTT
SPBPB2B2.01-R	GCTGCTCCCCCCCTATAAAG
SPBPB2B2.05-F	TGGTGGTGAATCGGTGCAT
SPBPB2B2.05-R	TTTTGGGAGCATTTGGATCAA
LTR_all_F	CGTACCATGTATGATACGATATGGAGA
LTR_all_R	AGCTCATAACTGAACTGAGGAACG
Proline_Probe	GCTGTTGTGGGAATCGAAC
Alanine_Probe	GGACAAGCCAGAACTCGAA
SPSNORNA.33_Probe	TATTTAGGCTCATTATGCAAGGA
ura4-5'-F	AGAGCTGAGGGGATGAAAAA
ura4-5'-R	TTTCGGATTTCTTCGTCAAA
ura4-3'-F	TGGTATCGGCTTGGATGTTA
ura4-3'-R	CAGCTCCATAGACTCCACGA
